# Estimating the effect of vaccination on antimicrobial-resistant typhoid fever in 73 countries supported by Gavi: a mathematical modelling study

**DOI:** 10.1016/S1473-3099(21)00627-7

**Published:** 2022-05

**Authors:** Ruthie Birger, Marina Antillón, Joke Bilcke, Christiane Dolecek, Gordon Dougan, Andrew J Pollard, Kathleen M Neuzil, Isabel Frost, Ramanan Laxminarayan, Virginia E Pitzer

**Affiliations:** a Department of Epidemiology of Microbial Diseases and Public Health Modeling Unit, Yale School of Public Health, Yale University, New Haven, CT, USA; b Swiss Tropical and Public Health Institute, Basel, Switzerland; c University of Basel, Basel, Switzerland; d Centre for Health Economics Research and Modelling Infectious Diseases, Vaccine and Infectious Disease Institute, University of Antwerp, Antwerp, Belgium; e Oxford Centre for Global Health Research, Centre for Tropical Medicine and Global Health, Nuffield Department of Medicine, University of Oxford, Oxford, UK; f Mahidol Oxford Tropical Medicine Research Unit, Faculty of Tropical Medicine, Mahidol University, Bangkok, Thailand; g Department of Medicine, University of Cambridge, Cambridge, UK; h Oxford Vaccine Group, University of Oxford, Oxford, UK; i National Institute for Health Research Oxford Biomedical Research Centre, Oxford, UK; j Center for Vaccine Development and Global Health, University of Maryland School of Medicine, Baltimore, MD, USA; k Center for Disease Dynamics, Economics & Policy, New Delhi, India; l High Meadows Environmental Institute, Princeton University, Princeton, NJ, USA; m Imperial College London, London, UK

## Abstract

**Background:**

Multidrug resistance and fluoroquinolone non-susceptibility (FQNS) are major concerns for the epidemiology and treatment of typhoid fever. The 2018 prequalification of the first typhoid conjugate vaccine (TCV) by WHO provides an opportunity to limit the transmission and burden of antimicrobial-resistant typhoid fever.

**Methods:**

We combined output from mathematical models of typhoid transmission with estimates of antimicrobial resistance from meta-analyses to predict the burden of antimicrobial-resistant typhoid fever across 73 lower-income countries eligible for support from Gavi, the Vaccine Alliance. We considered FQNS and multidrug resistance separately. The effect of vaccination was predicted on the basis of forecasts of vaccine coverage. We explored how the potential effect of vaccination on the prevalence of antimicrobial resistance varied depending on key model parameters.

**Findings:**

The introduction of routine immunisation with TCV at age 9 months with a catch-up campaign up to age 15 years was predicted to avert 46–74% of all typhoid fever cases in 73 countries eligible for Gavi support. Vaccination was predicted to reduce the relative prevalence of antimicrobial-resistant typhoid fever by 16% (95% prediction interval [PI] 0–49). TCV introduction with a catch-up campaign was predicted to avert 42·5 million (95% PI 24·8–62·8 million) cases and 506 000 (95% PI 187 000–1·9 million) deaths caused by FQNS typhoid fever, and 21·2 million (95% PI 16·4–26·5 million) cases and 342 000 (95% PI 135 000–1·5 million) deaths from multidrug-resistant typhoid fever over 10 years following introduction.

**Interpretation:**

Our results indicate the benefits of prioritising TCV introduction for countries with a high avertable burden of antimicrobial-resistant typhoid fever.

**Funding:**

The Bill & Melinda Gates Foundation.

## Introduction

Despite global improvements in sanitation and hygiene, typhoid fever, caused by the pathogen *Salmonella enterica* serovar Typhi (*S* Typhi), remains a major source of morbidity and mortality. Estimates of annual global cases of typhoid fever range from 10–20 million with 100 000 to 200 000 associated deaths. The majority of the burden is in low-income and middle-income countries, but incidence has been declining over the past three decades.^1^, ^2^, ^3^

Typhoid fever is treated with antibiotics, and the widespread prevalence of antimicrobial resistance is of growing concern. The most common forms of antimicrobial resistance for typhoid fever include reduced susceptibility to fluoroquinolones and multidrug resistance, defined as resistance to ampicillin, chloramphenicol, and co-trimoxazole, the three first-line drugs used to treat typhoid.^4^ Multidrug resistance has historically been prevalent in south Asia and southeast Asia, although there is evidence of decline since the 1990s.^5^, ^6^ No such decline has been observed in Africa, where prevalence of multidrug resistance is also high but variable, with outbreaks occurring throughout east Africa over the past 15 years.^5^, ^6^ Patterns for fluoroquinolone non-susceptibility (FQNS) differ from those for multidrug resistance, but generally there is high variability between countries and a global trend of increased FQNS.^5^, ^6^ Moreover, there are substantial gaps in the surveillance of antimicrobial resistance and typhoid fever in general.^2^, ^5^ First identified in 2016, an extensively drug-resistant strain of typhoid fever, exhibiting resistance to third-generation cephalosporins, in addition to ampicillin, chloramphenicol, co-trimoxazole, and fluoroquinolones, has emerged and spread in Pakistan, with sporadic cases reported in travellers throughout the world.^7^, ^8^, ^9^

Vaccination against typhoid fever could help avert antimicrobial resistance both directly, by preventing transmission of resistant infections, and indirectly, by preventing cases of infection caused by antimicrobial-susceptible *S* Typhi that would otherwise be treated with antibiotics and develop de novo resistance.^10^ In a 2019 clinical trial, prequalified typhoid conjugate vaccines (TCVs) have shown promising efficacy,^11^ and could offer a path towards decreasing global typhoid burden. Gavi, the Vaccine Alliance has pledged financial support for TCV introduction in eligible lower-income countries.^12^ Countries with a high burden of antimicrobial-resistant typhoid have been specifically prioritised for TCV introduction,^13^ and vaccines are being rolled out in Pakistan, Zimbabwe, and Liberia, but the effect of vaccination on antimicrobial resistance has yet to be determined.

**Research in context**

**Evidence before this study**

We searched PubMed and Web of Science using the terms “typhoid AND vaccin* AND (“antimicrobial resistan*” OR “drug resistan*”) AND model” for all studies published before Aug 9, 2021, without language restrictions. We excluded animal studies, reviews, and commentaries. Only one transmission modelling study has explicitly addressed the potential effect of vaccination on antimicrobial-resistant typhoid fever. This study was not specific to any particular setting, but instead focused on identifying the most influential model parameters affecting the prevalence of antimicrobial-resistant typhoid fever and the effect of vaccination. Moreover, the study only considered routine vaccination against typhoid fever. The overall effect of vaccination on antimicrobial-resistant typhoid fever depended on the relative fitness of resistant strains, the rate of recovery without treatment, and the prevalence of chronic carriers.

**Added value of this study**

We present country-specific estimates for the effect of typhoid conjugate vaccine (TCV) introduction on the burden of antimicrobial-resistant typhoid fever in 73 countries eligible for Gavi support. Model parameters reflect the best available data on typhoid incidence, forecasted vaccination coverage, and prevalence of fluoroquinolone non-susceptibility (FQNS) and multidrug resistance in each country. By incorporating age structure, age-specific vaccination, and assuming antimicrobial resistance emerged within the past 5–20 years, we predict that TCV introduction might lead to decreases in the relative prevalence of resistance and decreases in the overall burden of antimicrobial-resistant typhoid fever.

**Implications of all the available evidence**

Introduction of TCVs is becoming a reality in many countries with support from Gavi, which can have a substantial effect on the burden of antimicrobial-resistant typhoid fever. We find that routine vaccination of infants with TCV including a catch-up campaign up to age 15 years can avert approximately 53·5 million cases of antimicrobial-resistant typhoid fever over 10 years following introduction in 73 countries eligible for Gavi support. The avertable burden of FQNS is greatest in south Asia, whereas the avertable burden of multidrug resistance is greatest in sub-Saharan Africa. By reducing the incidence of typhoid fever, including both sensitive and resistant cases, TCV introduction is likely to lead to an overall reduction in the use of antibiotics—a knock-on effect that could reduce antibiotic selection pressure against a range of organisms. Curbing typhoid transmission can also prevent resistant plasmid transfer to other bacterial pathogens. Our results can help to inform country-level decision making and prioritise TCV introduction in countries with a high burden of antimicrobial-resistant typhoid fever.

Previous modelling studies have examined the potential effect of vaccination on typhoid fever. Although the model-predicted impact depends on factors such as the contribution of chronic carriers to transmission, vaccination programmes are likely to be cost-effective when incidence is high.^3^, ^14^, ^15^ A theoretical modelling study suggested that vaccination would decrease the overall incidence of antimicrobial-resistant typhoid, but not the proportion of cases with antimicrobial resistance. However, the model did not account for age structure and assumed antimicrobial resistance emerged over 100 years ago.^16^ Other studies have estimated that vaccination against *Streptococcus pneumoniae*, rotavirus, and *Mycobacterium tuberculosis* can potentially prevent millions of cases that would otherwise be treated with antibiotics, especially when paired with other interventions.^17^, ^18^

In this Article, we combine model predictions of the effect of TCV on typhoid fever with country-specific estimates of the burden of antimicrobial resistance (FQNS and multidrug resistance) to predict the number of antimicrobial-resistant typhoid fever cases, deaths, and disability-adjusted life years (DALYs) that can potentially be averted through TCV roll-out. The results can guide the prioritisation of TCV introduction in countries eligible for support from Gavi.

## Methods

### Study design

In our mathematical modelling study, to calculate the effect of TCV introduction on the antimicrobial-resistant typhoid burden we combined three pieces of information for each country: the effect of vaccination on total (sensitive and resistant) burden of typhoid fever, based on estimates of typhoid incidence and forecasted vaccine coverage; proportion of cases with FQNS and multidrug resistance; and the effect of vaccination on the proportion of cases that are drug resistant.

### The effect of vaccination on overall burden of typhoid

The predicted effect of vaccination over 10 years on cases, deaths, and DALYs was calculated for 54 countries eligible for Gavi support by Bilcke and colleagues;^3^ we extended the analysis to the 73 countries eligible for support in 2016. Our previous analysis indicated that whenever TCV introduction was cost-effective compared with no vaccination, routine vaccination at age 9 months plus a catch-up campaign up to age 15 years was the optimal strategy compared with routine vaccination alone or a more restricted catch-up campaign. Thus, in this study, we only considered routine vaccination with a catch-up campaign up to age 15 years.

To summarise the approach of Bilcke and colleagues,^3^ we used a transmission dynamic model to predict the effect of TCV. We categorised countries according to their demographic profile as “very young”, “young”, or “average” (appendix pp 2–4). For the corresponding demographic profile, we then sampled from a range of parameter estimates for the transmission rate as quantified by the basic reproductive number (R_0_) and proportion of typhoid infections that are symptomatic (*s*) to produce constant age-specific prevaccination incidence estimates that were consistent with uncertainty around the average age of typhoid fever cases and overall incidence in each country (appendix pp 2–3).^1^, ^2^ We also sampled from uncertainty in key model parameters likely to influence the predicted effect of vaccination, including the relative risk of infection for children younger than 2 years and those aged 2–4 years, and the relative infectiousness of chronic carriers.^3^, ^19^ The number of typhoid fever cases over time was predicted by simulating incidence 2000 times for each country and each vaccination scenario (ie, no vaccination, and routine vaccination at age 9 months with a catch-up campaign up to age 15 years) using: (1) parameters sampled from the posterior distributions of the prevaccination model fits; (2) estimates of typhoid natural history, vaccine effectiveness, and duration of protection that were common across all countries; and (3) Gavi forecasts of vaccine coverage assuming an unconstrained supply of TCVs (appendix pp 2–3). We assumed the initial vaccine efficacy was uniformly distributed between 80–95% and declined exponentially (appendix pp 2–3), consistent with data from clinical trials of Typbar TCV.^11^, ^20^ Parameter samples for the vaccination scenario were matched to those for the no-vaccination scenario when estimating the number of cases averted and associated uncertainty (95% prediction intervals [PIs]) in each country over a 10-year time horizon following TCV introduction. By modelling both incidence of clinical disease and transmission of infection, we were able to account for both the direct and indirect effects of vaccination. We did a post-hoc analysis to examine the drivers of country-to-country differences in the effect of vaccination (appendix p 12).

### Proportion of drug-resistant cases

We used the data from two systematic reviews to estimate the prevalence of FQNS and multidrug resistance.^5^, ^6^ We used the R meta package to combine estimates of prevalence of FQNS and multidrug resistance by country and Global Burden of Disease regions and super regions. There was significant heterogeneity in the prevalence of FQNS and multidrug resistance in almost all countries with multiple studies (*I*^2^=47–98%) and in almost all regions (*I*^2^=80–99%; appendix pp 5–8). Therefore, we used the random-effects estimates rather than pooled (fixed-effect) estimates of prevalence in most regions, where appropriate. We then estimated the parameters of the corresponding beta distributions to incorporate the uncertainty in the prevalence estimates of FQNS and multidrug resistance in our analysis (appendix pp 5–6).

Whenever possible, we used country-specific estimates of prevalence of antimicrobial resistance from all studies since 2010. For countries without published data on prevalence of antimicrobial resistance, we used the regional meta-analysis estimate and combined regions when a region only had studies from one country. However, there were regions with scarce or no data, so we made some region-specific decisions as described in the appendix (pp 5–6).

We assessed the effect of vaccination on the prevalence of FQNS and multidrug resistance separately. To generate estimates of the overall prevalence of antimicrobial resistance, we sampled independently from the beta distributions for FQNS and multidrug resistance and added the sampled values together; values greater than one were set to 100%. We did not estimate the overlap in FQNS and multidrug resistance; we also did not consider the prevalence of extensively drug-resistant typhoid (which is currently rare outside of Pakistan) in our analysis.

### The effect of vaccination on the proportion of cases that are drug resistant

To predict the potential effect of TCV introduction on the proportion of typhoid fever cases that are drug resistant, we modified a previously developed dynamic model for antimicrobial-resistant typhoid transmission to incorporate age structure and age-specific vaccination.^16^ The model accounts for important aspects of the natural history of typhoid infection, including the development of immunity to clinical disease and the chronic carrier state, and both acquired and transmitted resistance (appendix pp 9–11).

To explore uncertainty around possible changes to the proportion of cases that are resistant after vaccination, we ran the model using a range of plausible values for R_0_, the proportion of infections that are symptomatic, vaccination coverage, and parameters related to resistance, including the rate of treatment-induced resistance, the relative transmissibility of the resistant strain, and the duration of primary and subclinical resistant infections (table 1). We introduced resistance 10 years before vaccination by setting the resistance parameters *(P, r*_R_*,* δ_1R_*,* δ_2R_*)* to non-zero values after that timepoint. We then calculated the proportion of cases resistant for each of the 10 years after TCV roll-out under the baseline assumption of no vaccination and assuming routine vaccination plus a catch-up campaign up to age 15 years.

**Table 1:** Input parameters varied in the model to predict the effect of vaccination on the proportion of cases that are drug resistant

	**Description**	**Values**	**Source**
**Parameter**			
R_0_	Basic reproductive number	1·5, 2·5, 3·5, 7, 10·5	Bilcke et al (2019)^3^
s	Proportion symptomatic	0·01, 0·05, 0·10, 0·5	Bilcke et al (2019)^3^
κ	Vaccination coverage (increase over 10 years)	70–79%, 62–80%, 86–95%	Bilcke et al (2019)^3^
**Resistance parameters**			
ρ	Rate of resistance acquisition from treatment	0·01–5 per week	Assumption
r_R_	Relative risk of transmission for resistant strain	0·3–3	Assumption and Baker et al (2013)^26^
δ_1R_	Rate of recovery from primary infection with a resistant strain	0·075–0·7 per week	Assumption and Hornick et al (1970)^27^
δ_2R_	Rate of recovery from subclinical infection with a resistant strain	0·075–0·7 per week	Assumption and Hornick et al (1970)^27^

The four resistance parameters govern the prevalence of resistance; we selected values from within a reasonable range for each parameter and used Latin-hypercube sampling from the uniform range of each parameter to produce model outputs, which resulted in a wide range of initial resistance prevalence.

We summarised the effect of vaccination on the prevalence of antimicrobial resistance by estimating the proportional difference between the TCV versus no-vaccination scenario for each parameter set (appendix p 13). We then calculated the median and 95% PI of these estimates to get a distribution for the effect of vaccination on the prevalence of antimicrobial resistance across settings. We assumed that the effect of vaccination on FQNS and multidrug resistance would be similar.

We examined the association between the predicted change in the proportion resistant and each of the varied parameters unrelated to resistance (R_0_, proportion symptomatic, and vaccination coverage). Since the model with drug resistance was not country-specific, we developed a linear regression model on the basis of these factors to characterise how changes to the proportion resistant should vary across countries (appendix pp 13–15). We also did a sensitivity analysis to examine how the predicted change in the relative prevalence of antimicrobial resistance varied depending on when resistance emerged (5–20 years before vaccine introduction; appendix p 9).

### Deaths and DALYs

Estimates of deaths and DALYs lost due to typhoid fever were calculated by applying case fatality risk estimates, disability weights, and life-expectancy estimates to the predicted number of typhoid cases stratified by age and time. Available country-specific estimates of the case fatality risk and life-expectancy were used.^3^ To extend the analysis to 19 countries eligible for Gavi support before 2018, we used available country-specific estimates, but otherwise used a common meta-analysis estimate for all countries. We assumed that the probability of death and years of life lived with disability were one to three times (sampled as a uniform random variable) the corresponding values for sensitive cases.^3^ We sampled from the updated distributions for prevalence of antimicrobial resistance in each country.

To obtain estimates of the number of antimicrobial-resistant typhoid fever cases, deaths, and DALYs averted by vaccination for each country, we first ran 2000 simulations of the transmission model under the baseline scenario of no vaccination, sampling from the full range of model parameters. We then ran 2000 simulations of the routine TCV introduction with the catch-up campaign scenario, using the corresponding parameter samples for the no-vaccination scenario and multiplying the total prevalence estimates of antimicrobial resistance by the change in the proportion resistant calculated from the linear regression model (ie, based on the sampled values of R_0_ and the proportion symptomatic). We calculated cases, deaths, and DALYs averted by subtracting the results of the TCV simulations from the baseline no-vaccination simulations. To generate estimates for the FQNS and multidrug resistance burden averted, we repeated this process using the estimated prevalence of FQNS and multidrug resistance for each country. No discounting of future outcomes was applied.

### Role of the funding source

The funder had no role in study design, data collection, data analysis, data interpretation, or writing of the report.

## Results

For the 73 countries eligible for Gavi support, implementing routine use of TCV with a catch-up campaign up to age 15 years was predicted to avert 66·7 million (95% PI 48·1–88·3 million) cases of typhoid fever over 10 years, reflecting reductions on the order of 46–74% in individual countries (range of means; table 2). The predicted vaccine effect was lower in countries with an older population and lower expected vaccine coverage among the age-eligible population (appendix p 12).

**Table 2:** Predicted number of typhoid cases, deaths, and DALYs averted over 10 years following introduction of routine immunisation with typhoid conjugate vaccines plus a catch-up campaign

	**Baseline total typhoid cases (thousands)**	**Baseline total typhoid deaths**	**Baseline total typhoid DALYs (thousands)**	**Total typhoid cases averted (thousands)**	**Total typhoid deaths averted**	**Total typhoid DALYs averted (thousands)**	**Percentage of typhoid cases averted**
Afghanistan	2298 (336–4 301)	9681 (346–182 786)	509 (23–9574)	1353 (194–2982)	6153 (202–116 215)	330 (13–6032)	66% (35–82%)
Angola	546 (300–880)	2261 (115–37 957)	91 (7–1529)	324 (145–633)	1444 (68–23 819)	59 (4–986)	66% (37–82%)
Armenia	2 (0·203–4)	9 (0–192)	0·539 (0·022–11)	1 (0·094–3)	5 (0–102)	0·310 (0·012–6)	53% (30–75%)
Azerbaijan	20 (1–40)	80 (2–1714)	4 (0·165–89)	10 (0·713–27)	47 (1–1048)	3 (0·099–58)	60% (34–82%)
Bangladesh	14 083 (9526–18 559)	26 812 (428–1 232 180)	1723 (80–75 304)	8508 (4066–13 489)	17 390 (253–778 865)	1153 (50–48 566)	66% (34–83%)
Benin	1230 (249–2222)	4260 (175–76 137)	222 (12–4066)	751 (156–1639)	2789 (108–47 662)	149 (7–2495)	70% (35–86%)
Bhutan	20 (8–32)	96 (5–1705)	5 (0·367–94)	11 (4–21)	52 (3–927)	3 (0·200–52)	57% (29–76%)
Bolivia	199 (14–397)	530 (12–9845)	29 (0·827–508)	112 (8–279)	314 (6–5825)	18 (0·443–321)	61% (32–81%)
Burkina Faso	975 (797–1177)	2873 (167–37 619)	141 (10–1820)	692 (360–951)	1990 (109–25712)	101 (7–1305)	72% (39–87%)
Burundi	1268 (184–2370)	4938 (182–87 907)	234 (10–4094)	857 (125–1842)	3434 (129–61654)	170 (8–2956)	74% (40–88%)
Cambodia	808 (573–1061)	4046 (208–61 178)	232 (15–3375)	479 (237–748)	2502 (131–37 356)	149 (10–2174)	65% (34–81%)
Cameroon	557 (454–674)	2385 (122–35666)	115 (8–1715)	381 (184–529)	1659 (85–24332)	82 (5–1159)	72% (37–87%)
Central African Republic	250 (62–455)	917 (38–15420)	38 (2–611)	149 (35–327)	579 (23–10075)	24 (1–412)	66% (34–82%)
Chad	864 (380–1385)	2578 (136–35707)	107 (8–1468)	529 (188–1015)	1633 (81–23366)	71 (5–986)	68% (33–83%)
Comoros	6 (5–7)	26 (1–375)	1 (0·095–19)	4 (2–6)	19 (1–279)	0·996 (0·069–14)	74% (44–89%)
Congo (Brazzaville)	106 (33–195)	381 (20–6760)	19 (1–349)	71 (21–154)	266 (13–4832)	14 (0·905–249)	73% (43–88%)
Côte d'Ivoire	916 (364–1463)	2958 (171–50 474)	133 (9–2221)	577 (194–1102)	1986 (105–34475)	92 (6–1520)	72% (37–87%)
Cuba	18 (1–35)	46 (1–906)	3 (0·094–52)	7 (0·555–20)	20 (1–427)	1 (0·053–29)	46% (25–71%)
Democratic Republic of the Congo	7054 (1189–13 968)	26 623 (1125–467 392)	1279 (66–22427)	4209 (711–9669)	16 903 (640–280 899)	837 (41–13627)	65% (38–80%)
Djibouti	8 (7–10)	36 (2–542)	2 (0·137–29)	5 (2–7)	22 (1–328)	1 (0·082–18)	63% (32–80%)
Eritrea	185 (79–299)	778 (37–12 134)	42 (2–628)	124 (46–237)	562 (26–8332)	30 (2–449)	74% (44–88%)
Ethiopia	3823 (1419–6393)	42 903 (1949–683 400)	2384 (121–38 973)	2114 (674–4219)	25576 (1140–397 863)	1470 (78–22 977)	63% (33–78%)
Georgia	2 (0·254–5)	11 (0–199)	0·583 (0·021–11)	1 (0·109–3)	5 (0–98)	0·314 (0·011–6)	50% (27–73%)
Ghana	614 (530–717)	2737 (147–40 059)	137 (10–1936)	434 (231–595)	1944 (102–28 230)	101 (7–1457)	73% (43–88%)
Guinea	548 (232–889)	2065 (88–34 156)	101 (6–1615)	340 (120–651)	1355 (57–22 851)	68 (4–1112)	68% (35–83%)
Guinea-Bissau	32 (7–57)	109 (6–1838)	5 (0·303–79)	21 (4–44)	75 (4–1257)	3 (0·205–57)	73% (42–87%)
Guyana	6 (0·511–11)	16 (0–285)	0·845 (0·032–14)	3 (0·256–7)	8 (0–139)	0·460 (0·016–8)	52% (27–74%)
Haiti	119 (12–245)	338 (10–5880)	15 (0·659–253)	55 (6–149)	164 (5–3119)	8 (0·320–156)	50% (27–73%)
Honduras	55 (8–107)	153 (5–2632)	9 (0·319–154)	31 (4–72)	89 (3–1668)	6 (0·199–102)	60% (33–81%)
India	41 362 (12 871–69 611)	391 952 (75 714–1 862 894)	22 126 (4364–105 476)	19 503 (5409–39 629)	202 445 (37 807–1 051 607)	12 006 (2291–61 292)	54% (32–69%)
Indonesia	5458 (2176–8891)	6126 (1291–28 386)	377 (85–1652)	2556 (921–4994)	2932 (600–14 595)	186 (40–901)	51% (26–66%)
Kenya	1035 (891–1183)	7719 (494–96 294)	389 (29–4593)	709 (373–957)	5275 (306–65 270)	275 (20–3287)	72% (42–86%)
Kiribati	4 (2–7)	18 (1–314)	1 (0·071–18)	2 (0·838–5)	11 (1–182)	0·655 (0·043–10)	65% (33–81%)
Kyrgyzstan	6 (0·419–13)	25 (1–560)	1 (0·051–29)	3 (0·228–9)	16 (0–327)	0·929 (0·031–19)	63% (36–82%)
Laos	418 (246–593)	590 (21–14015)	35 (3–782)	238 (105–409)	353 (13–9194)	22 (1–525)	63% (32–79%)
Lesotho	6 (0·865–11)	19 (1–342)	0·764 (0·040–13)	3 (0·501–7)	12 (0–225)	0·511 (0·026–9)	65% (35–82%)
Liberia	390 (99–713)	1460 (68–26795)	70 (4–1225)	250 (65–558)	985 (44–17825)	49 (3–858)	70% (41–87%)
Madagascar	1907 (374–3405)	7312 (311–130 539)	397 (21–7034)	1157 (220–2484)	4913 (198–82 656)	272 (14–4469)	69% (38–84%)
Malawi	251 (170–336)	989 (49–13 960)	52 (3–727)	169 (86–267)	677 (35–10 045)	36 (2–526)	72% (41–87%)
Mali	559 (352–797)	1767 (104–23 836)	86 (6–1150)	379 (178–621)	1221 (64–17 280)	60 (4–828)	72% (39–87%)
Mauritania	86 (73–100)	275 (15–3615)	14 (1–183)	59 (31–79)	180 (10–2468)	10 (0·666–133)	70% (38–85%)
Moldova	1 (0·076–2)	5 (0–123)	0·282 (0·008–6)	0·581 (0·035–2)	3 (0–62)	0·164 (0·004–3)	54% (29–74%)
Mongolia	53 (3–112)	224 (5–4698)	12 (0·307–229)	28 (2–75)	127 (3–2609)	7 (0·178–145)	59% (34–81%)
Mozambique	712 (517–942)	2837 (161–42 263)	129 (10–1884)	497 (257–749)	2008 (105–29 791)	93 (6–1325)	74% (43–88%)
Myanmar	1406 (1112–1695)	6237 (345–92 882)	331 (24–4954)	745 (355–1127)	3443 (179–53 785)	195 (12–2979)	57% (30–75%)
Nepal	847 (364–1346)	3735 (190–59 846)	222 (15–3541)	492 (169–944)	2399 (110–37 664)	146 (9–2294)	65% (35–81%)
Nicaragua	47 (5–96)	123 (5–2247)	7 (0·339–129)	25 (2–66)	71 (3–1200)	4 (0·206–74)	59% (32–80%)
Niger	651 (374–991)	2605 (136–37 836)	135 (9–2045)	435 (193–764)	1804 (92–26 416)	95 (6–1390)	72% (40–87%)
Nigeria	8463 (4305–13 193)	147 422 (12 331–1 762 558)	5808 (539–70 849)	4548 (1928–8488)	85667 (7170–1 065 947)	3553 (314–44 268)	60% (36–76%)
North Korea	657 (52–1267)	1807 (67–37 045)	100 (5–2026)	318 (26–790)	987 (34–20 168)	60 (2–1188)	54% (28–74%)
Pakistan	4205 (2522–6021)	7838 (860–69 350)	465 (68–3949)	1979 (869–3391)	4028 (410–37 056)	245 (32–2147)	54% (28–69%)
Papua New Guinea	1520 (359–1667)	6028 (301–94 164)	334 (21–5091)	836 (201–1151)	3206 (153–53 604)	183 (11–2929)	57% (24–74%)
Rwanda	286 (138–448)	1236 (64–20 914)	66 (4–1106)	192 (82–363)	867 (45–15 332)	48 (3–797)	74% (43–88%)
São Tomé and Príncipe	2 (0·713–3)	6 (0–92)	0·325 (0·022–5)	1 (0·403–2)	4 (0–66)	0·238 (0·015–4)	72% (44–88%)
Senegal	242 (185–329)	746 (55–9350)	39 (4–491)	164 (87–265)	523 (36–6541)	29 (3–365)	71% (44–88%)
Sierra Leone	403 (153–654)	1596 (76–23 794)	68 (4–957)	273 (95–521)	1145 (52–17 710)	49 (3–703)	74% (42–88%)
Solomon Islands	49 (11–88)	196 (10–3130)	12 (0·720–185)	32 (8–68)	140 (6–2166)	9 (0·511–130)	74% (39–88%)
Somalia	431 (252–689)	1867 (102–29 095)	77 (6–1176)	253 (118–489)	1164 (62–17 965)	50 (4–792)	64% (36–82%)
South Sudan	265 (170–376)	1183 (62–18 950)	52 (4–810)	145 (65–247)	680 (35–11 389)	31 (2–490)	60% (32–76%)
Sri Lanka	188 (42–329)	808 (36–13 554)	48 (3–837)	94 (20–209)	436 (19–8229)	28 (2–500)	57% (31–76%)
Sudan	662 (241–1101)	2753 (126–44 516)	152 (9–2442)	423 (140–856)	1878 (82–29 031)	106 (6–1644)	73% (39–88%)
Tajikistan	12 (1–25)	51 (1–982)	3 (0·100–54)	7 (0·714–18)	35 (1–731)	2 (0·071–42)	71% (42–88%)
Tanzania	1955 (777–3279)	7691 (344–117 287)	430 (26–6701)	1321 (472–2576)	5432 (249–89 376)	312 (19–4894)	74% (43–88%)
The Gambia	35 (25–46)	150 (8–2061)	7 (0·494–100)	24 (13–38)	108 (5–1406)	6 (0·345–72)	74% (44–88%)
Timor-Leste	42 (33–53)	187 (9–2639)	11 (0·754–157)	28 (13–41)	125 (6–1745)	8 (0·479–107)	70% (36–86%)
Togo	161 (129–196)	680 (36–10 021)	34 (2–483)	114 (60–158)	503 (25–7019)	25 (2–360)	74% (42–88%)
Uganda	532 (434–641)	2331 (124–34 593)	116 (8–1654)	375 (193–518)	1670 (90–24 335)	86 (6–1234)	74% (42–88%)
Ukraine	16 (1–31)	70 (2–1365)	4 (0·153–70)	7 (0·531–18)	34 (1–665)	2 (0·084–38)	51% (29–74%)
Uzbekistan	30 (2–62)	124 (4–2474)	6 (0·244–131)	16 (1–43)	71 (2–1457)	4 (0·147–83)	60% (35–82%)
Vietnam	1008 (602–1437)	18 016 (2391–131 262)	1080 (144–7668)	511 (219–913)	9833 (1166–73 354)	627 (74–4693)	56% (30–76%)
Yemen	7338 (602–8860)	18 010 (537–271 293)	1033 (43–15 369)	3924 (346–6342)	10 059 (324–141 892)	599 (24–8373)	62% (26–82%)
Zambia	431 (246–665)	1657 (91–25 197)	82 (6–1246)	293 (134–532)	1186 (59–18 325)	61 (4–924)	73% (42–88%)
Zimbabwe	233 (16–485)	1203 (35–18 952)	56 (2–863)	157 (11–377)	866 (25–13 274)	41 (1–627)	73% (42–88%)

Data are n (PI) or % (PI). Total cases include both antimicrobial-resistant and antimicrobial-sensitive typhoid fever cases. The total number of deaths and DALYs at baseline and averted by vaccination depend on the prevalence of antimicrobial resistance, since we assume that the risk of death and years lived with disability are one to three times higher for resistant cases compared with sensitive typhoid fever cases· Countries are listed alphabetically. DALYs=disability-adjusted life-years. PI=prediction interval.

**Figure 1:**
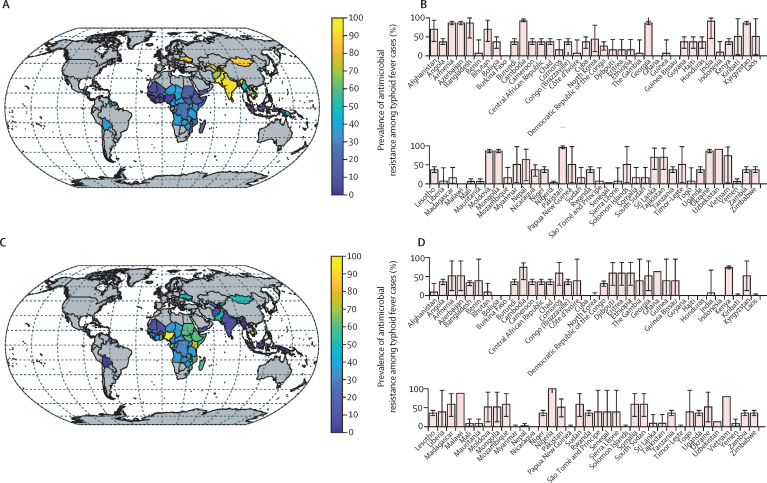
Estimated prevalence of antimicrobial resistance among typhoid fever cases in 73 countries eligible for Gavi support (A) Mean prevalence of fluoroquinolone non-susceptibility for each country. (B) Bar plots of the mean prevalence and 95% CI of fluoroquinolone non-susceptibility for each country. (C) Mean prevalence of multidrug resistance for each country. (D) Bar plots of the mean prevalence and 95% CI of multidrug resistance for each country. Data sources are listed in the appendix (pp 2, 5–6).

The prevalence of FQNS tended to be high in Asia and lower (<50%) in other parts of the world, whereas prevalence of multidrug resistance varied from country to country but was more common in central and eastern sub-Saharan Africa (figure 1). On the basis of the updated estimates of prevalence of antimicrobial resistance, we predicted that TCV introduction including a catch-up campaign up to age 15 years would avert 826 000 deaths (381 000–2·4 million) and 44·4 million DALYs (18·7–143 million) caused by typhoid fever over 10 years following introduction (table 2).

When we simulated the updated transmission model incorporating both age structure and drug-resistant states, the resulting set of 1200 simulations yielded a mean reduction in the relative proportion resistant of 16·1% (95% PI 0–49; appendix p 13). We estimated that the relative prevalence of drug resistance following vaccine introduction decreased by 1·1% for each one unit increase in R_0_ and by 0·83% for each 0·1 increase in the proportion symptomatic, but did not vary depending on the vaccine coverage (appendix p 14). When resistance emerges earlier (20 years before vaccine introduction), the postvaccination change in proportion resistant was slightly reduced (10·1%, 95% PI 2–37), whereas when resistance emerges later (5 years before vaccine introduction), the change in proportion resistant was slightly greater (19·1%, 95% PI 0–53; appendix p 13).

Introduction of routine TCV vaccination with a catch-up campaign up to age 15 years was predicted to avert 42·5 million (95% PI 24·8–62·9 million) cases of typhoid fever with FQNS and 21·2 million (95% PI 16·4–26·5 million) cases with multidrug resistance over 10 years (appendix pp 16–21). Overall, 53·5 million (95% PI 37·3–75·3 million) cases of antimicrobial-resistant typhoid fever could be prevented (table 3). The avertable burden of FQNS was greatest in south Asia, whereas the avertable burden of multidrug resistance was greatest in sub-Saharan Africa. Nearly half of the avertable cases of typhoid with FQNS were in India (21·1 million, 95% PI 6·3–41·3 million), whereas the greatest number of avertable cases with multidrug resistance occurred in Nigeria (5·2 million, 95% PI 2·4–9·3 million); country-specific estimates of cases, deaths, and DALYs averted are available in the appendix (pp 16–21).

**Table 3:** Predicted number of antimicrobial-resistant typhoid cases, deaths, and DALYs averted over 10 years following the introduction of routine immunisation with typhoid conjugate vaccines plus a catch-up campaign up to age 15 years

	**Baseline total antimicrobial-resistant cases (thousands)**	**Baseline total antimicrobial-resistant deaths**	**Baseline total antimicrobial-resistant DALYs (thousands)**	**Total antimicrobial-resistant cases averted (thousands)**	**Total antimicrobial-resistant deaths averted**	**Total antimicrobial-resistant DALYs averted (thousands)**
**Sub–Saharan Africa**						
Angola	350 (1–807)	1265 (3–36 599)	50 (0·113–1487)	238 (0·663–597)	867 (2–27 557)	37 (0·094–1116)
Benin	520 (58–1691)	2601 (64–63 788)	130 (3–3254)	366 (38–1241)	1707 (42–45 100)	92 (3–2367)
Burkina Faso	53 (8–165)	269 (9–6232)	13 (0·413–297)	39 (6–130)	200 (6–4980)	10 (0·406–239)
Burundi	861 (118–1730)	3832 (144–79948)	177 (6–3841)	619 (84–1351)	2778 (98–59 205)	138 (6–2871)
Cameroon	427 (2–652)	1517 (6–41939)	70 (0·283–1903)	293 (2–520)	1082 (5–29 397)	54 (0·241–1429)
Central African Republic	115 (12–348)	536 (14–13346)	21 (0·557–528)	78 (8–253)	365 (9–9341)	16 (0·536–377)
Chad	124 (39–330)	651 (24–15151)	27 (1–610)	87 (26–247)	450 (17–10 438)	20 (1–444)
Comoros	4 (3–6)	21 (1–392)	1 (0·049–20)	3 (2–5)	16 (1–306)	0·848 (0·054–16)
Congo (Brazzaville)	47 (6–142)	233 (6–5319)	11 (0·298–263)	36 (4–112)	174 (4–3963)	9 (0·288–202)
Côte d'Ivoire	507 (2–1397)	1824 (4–40471)	78 (0·176–1739)	363 (1–1075)	1284 (3–31 048)	60 (0·151–1383)
Democratic Republic of the Congo	4270 (710–8603)	19 696 (761–443907)	915 (35–21120)	2801 (475–6337)	13 160 (490–295 868)	655 (30–14478)
Djibouti	5 (4–7)	29 (1–550)	1 (0·071–29)	4 (2–5)	18 (1–366)	0·993 (0·065–19)
Eritrea	125 (50–220)	617 (28–12 032)	32 (1–628)	92 (34–178)	464 (20–9039)	25 (1–473)
Ethiopia	2546 (922–4715)	31 974 (1442–599 487)	1757 (80–33461)	1588 (544–3346)	20637 (934–390 554)	1181 (57–22 235)
Ghana	398 (282–518)	2135 (100–37 021)	105 (5–1840)	301 (177–435)	1583 (71–28 413)	82 (5–1407)
Guinea	255 (34–688)	1240 (37–27811)	59 (2–1326)	177 (24–517)	850 (26–18 969)	43 (2–953)
Guinea Bissau	13 (1–43)	63 (2–1584)	3 (0·069–67)	10 (0·956–34)	47 (1–1186)	2 (0·079–51)
Kenya	821 (674–978)	7363 (397–96 055)	362 (20–4899)	612 (388–820)	5336 (274–69338)	274 (18–3630)
Lesotho	2 (0·304–7)	11 (0–278)	0·426 (0·011–11)	2 (0·213–5)	8 (0–195)	0·307 (0·011–8)
Liberia	210 (0·654–665)	790 (2–25996)	37 (0·102–1254)	147 (0·463–517)	555 (2–20213)	28 (0·081–1010)
Madagascar	1269 (237–2448)	5781 (213–113423)	307 (11–5945)	866 (169–1854)	4080 (156–79 351)	225 (11–4344)
Malawi	115 (31–238)	567 (22–11538)	29 (1–588)	86 (22–185)	419 (17–8687)	22 (1–460)
Mali	84 (30–191)	446 (18–9372)	21 (0·789–436)	63 (21–151)	331 (12–6886)	16 (0·845–334)
Mauritania	13 (5–28)	71 (3–1489)	4 (0·147–75)	10 (4–21)	50 (2–1021)	3 (0·144–54)
Mozambique	331 (88–669)	1689 (63–34562)	72 (3–1538)	248 (65–536)	1249 (49–24 431)	57 (3–1159)
Niger	330 (49–767)	1634 (58–30197)	83 (3–1548)	241 (37–603)	1175 (38–23 139)	63 (3–1213)
Nigeria	8358 (4282–13043)	155 553 (10371–1687005)	6128 (406–674 44)	5236 (2438–9256)	99 939 (6524–10 741 66)	4130 (283–43 283)
Rwanda	193 (86–333)	999 (41–18844)	51 (2–992)	143 (60–275)	749 (33–14371)	41 (2–767)
São Tomé and Príncipe	0·758 (0·105–2)	4 (0–84)	0·195 (0·006–4)	0·571 (0·075–2)	3 (0–63)	0·152 (0·006–3)
Senegal	175 (0·514–302)	435 (1–8862)	22 (0·069–451)	119 (0·350–247)	328 (1–6764)	18 (0·059–350)
Sierra Leone	226 (0·778–611)	877 (3–28004)	35 (0·105–1091)	166 (0·597–493)	647 (2–19594)	28 (0·101–794)
Somalia	294 (153–502)	1563 (68–28375)	64 (3–1137)	194 (88–377)	1027 (46–20274)	45 (3–865)
South Sudan	178 (107–279)	944 (43–18246)	41 (2–771)	114 (57–200)	595 (27–11538)	27 (2–496)
Tanzania	1048 (370–2370)	5531 (236–115361)	291 (13–6194)	778 (258–1907)	4143 (170–85127)	235 (12–4762)
The Gambia	25 (0·064–43)	81 (0–2366)	4 (0·009–116)	18 (0·052–36)	61 (0–1823)	3 (0·008–94)
Togo	117 (0·602–187)	410 (1–10287)	20 (0·061–490)	84 (0·460–155)	307 (1–7703)	16 (0·057–372)
Uganda	356 (242–490)	1884 (82–34077)	91 (4–1635)	273 (154–399)	1401 (60–25100)	71 (5–1240)
Zambia	193 (58–446)	1000 (33–20945)	48 (2–1006)	143 (40–362)	747 (25–15518)	37 (2–756)
Zimbabwe	100 (6–311)	697 (18–13592)	31 (0·807–605)	73 (5–246)	531 (13–9896)	25 (0·669–472)
Subtotal	25 513 (18 532–34 237)	309 172 (27 221–3 281 008)	13 950 (1335–145 451)	17 391 (10 172–25 125)	200 986 (18 106–2 173 672)	9344 (887–99 585)
**North Africa and the Middle East**						
Afghanistan	1990 (290–3782)	9033 (305–215 241)	459 (16–10 899)	1308 (192–2755)	5902 (194–145 554)	311 (14–7604)
Sudan	438 (154–818)	2156 (91–39 870)	116 (5–2137)	317 (103–647)	1578 (61–27 789)	89 (5–1593)
Yemen	946 (80–2229)	4179 (136–93 900)	236 (8–5425)	509 (42–1468)	2281 (60–55 097)	137 (4–3327)
Subtotal	3384 (1276–5690)	17 173 (705–324 829)	950 (53–18 068)	2173 (718–4020)	10 949 (493–215 004)	603 (35–11 753)
**Central Europe, eastern Europe, and central Asia**						
Armenia	2 (0·191–4)	9 (0–205)	0·505 (0·014–12)	1 (0·108–3)	5 (0–129)	0·325 (0·011–8)
Azerbaijan	18 (1–38)	79 (2–1952)	4 (0·097–101)	11 (0·803–28)	50 (1–1250)	3 (0·073–69)
Georgia	2 (0·239–4)	10 (0–247)	0·548 (0·017–13)	1 (0·134–3)	6 (0–133)	0·337 (0·012–8)
Kyrgyzstan	6 (0·394–12)	25 (1–550)	1 (0·034–31)	4 (0·253–9)	17 (0–378)	0·956 (0·028–21)
Moldova	1 (0·071–2)	5 (0–117)	0·274 (0·006–6)	0·682 (0·038–2)	3 (0–70)	0·177 (0·004–4)
Tajikistan	11 (1–25)	48 (1–1101)	3 (0·075–60)	8 (0·757–20)	36 (1–784)	2 (0·074–46)
Ukraine	15 (1–30)	66 (2–1533)	3 (0·095–77)	8 (0·686–20)	35 (1–865)	2 (0·069–49)
Uzbekistan	27 (2–58)	119 (3–2772)	6 (0·139–136)	17 (1–43)	78 (2–1869)	4 (0·117–97)
Subtotal	85 (41–133)	430 (22–7807)	23 (2–411)	54 (24–96)	274 (13–5382)	15 (1–292)
**South Asia**						
Bangladesh	12 678 (8127–17 436)	30 145 (421–1 504 548)	1846 (25–93 708)	8366 (4299–13 323)	19 359 (276–996 718)	1275 (54–62 999)
Bhutan	18 (7–28)	89 (4–1798)	5 (0·209–95)	10 (4–19)	51 (2–1036)	3 (0·160–60)
India	36 679 (11 467–63 526)	366 429 (66 524–1 833 006)	20 573 (3783–103 446)	21 146 (6162–41 246)	215 352 (36 349–1 050 835)	12 597 (2186–62 134)
Nepal	657 (278–1107)	3375 (138–60 693)	195 (8–3583)	435 (165–827)	2195 (94–40 660)	138 (8–2497)
Pakistan	3445 (1884–5436)	7104 (756–63 495)	403 (41–3520)	1961 (959–3487)	4058 (418–36 332)	247 (32–2091)
Subtotal	54 036 (26 825–81 427)	495 751 (95 860–2 787 372)	28 949 (5362–159 263)	32 220 (14 179–54 393)	299 058 (52 391–1 762 004)	17 796 (3050–108 401)
**Southeast Asia, east Asia, and Oceania**						
Cambodia	713 (452–1007)	3801 (170–69 238)	213 (9–3955)	472 (242–759)	2428 (104–46 722)	150 (8–2654)
Indonesia	132 (15–552)	291 (21–2363)	16 (1–136)	76 (9–348)	168 (12–1366)	10 (0·781–85)
Kiribati	3 (0·478–6)	13 (0–279)	0·698 (0·024–15)	2 (0·305–4)	8 (0–182)	0·485 (0·020–11)
Laos	276 (63–528)	424 (10–13208)	23 (0·530–738)	177 (37–371)	263 (7–8425)	16 (0·854–480)
Mongolia	49 (3–106)	198 (6–4821)	10 (0·282–242)	31 (2–78)	125 (3–3100)	7 (0·241–171)
Myanmar	1001 (189–1567)	4585 (172–86 873)	240 (9–4479)	578 (112–1066)	2717 (98–53 723)	154 (7–2890)
North Korea	149 (11–519)	677 (16–18 534)	37 (0·902–1025)	90 (7–328)	409 (11–10534)	24 (0·670–608)
Papua New Guinea	1022 (150–1546)	4571 (150–91 792)	244 (8–5114)	499 (61–1021)	2274 (60–48 868)	131 (5–2716)
Solomon Islands	32 (5–77)	141 (4–3351)	8 (0·250–201)	23 (3–60)	106 (3–2563)	6 (0·219–158)
Sri Lanka	163 (37–289)	754 (28–16 936)	45 (2–999)	97 (21–205)	470 (17–10539)	30 (1–643)
Timor-Leste	30 (7–48)	141 (6–3079)	9 (0·331–185)	21 (5–37)	98 (4–2118)	6 (0·314–130)
Vietnam	676 (128–1281)	14 044 (1146–122 159)	833 (71–7088)	404 (78–874)	8492 (698–70 495)	529 (43–4581)
Subtotal	4241 (2852–5584)	36 995 (3892–384 680)	2119 (245–21 601)	2553 (1361–3830)	21 888 (2080–242 140)	1302 (136–14 263)
**Latin America and the Caribbean**						
Bolivia	27 (2–103)	129 (3–3577)	7 (0·144–193)	17 (1–71)	83 (2–2473)	5 (0·126–138)
Cuba	2 (0·179–10)	12 (0–299)	0·659 (0·015–16)	1 (0·095–6)	6 (0–159)	0·392 (0·010–10)
Guyana	0·831 (0·073–3)	4 (0–99)	0·190 (0·005–5)	0·485 (0·041–2)	2 (0–61)	0·123 (0·004–3)
Haiti	18 (1–65)	85 (2–2152)	4 (0·075–94)	10 (0·859–41)	49 (1–1389)	2 (0·064–65)
Honduras	8 (0·920–28)	38 (1–873)	2 (0·054–50)	5 (0·567–20)	25 (1–607)	2 (0·044–37)
Nicaragua	7 (0·537–25)	31 (1–936)	2 (0·051–54)	4 (0·364–17)	20 (1–607)	1 (0·042–37)
Subtotal	72 (27–162)	374 (17–7300)	20 (1–380)	45 (16–111)	235 (10–4757)	13 (1–258)
Total	87 753 (59 981–116 333)	1 216 643 (463 630–3 534 868)	61 456 (24 655–201 446)	53 484 (37 262–75 335)	818 489 (324 752–2 753 200)	41 611 (17 607–116 891)

Data are n (PI). To estimate the total prevalence of antimicrobial-resistant typhoid fever, we assume that the prevalence of fluoroquinolone non-susceptibility and multidrug resistance are independent in each country and sum the sampled prevalence for each iteration of the model. Countries are grouped by Global Burden of Disease regions and super regions. DALYs=disability-adjusted life-years. PI=prediction interval.

**Figure 2:**
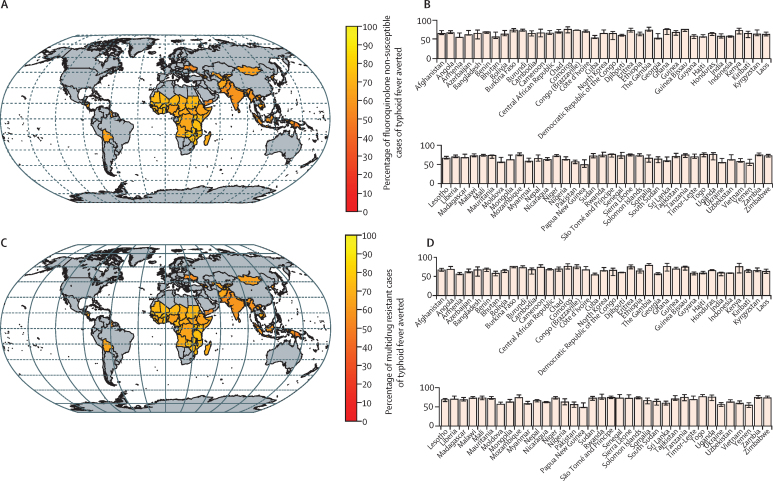
Predicted effect of vaccination on the incidence of fluoroquinolone non-susceptible and multidrug-resistant cases of typhoid fever (A) Percentage of fluoroquinolone non-susceptible cases of typhoid fever averted over 10 years following introduction of routine immunisation with typhoid conjugate vaccines plus a catch-up campaign for each country. (B) Bar plots of the mean reduction and 95% prediction interval of fluoroquinolone non-susceptibility for each country. (C) Percentage of multidrug resistant cases of typhoid fever averted over 10 years following introduction of routine immunisation with typhoid conjugate vaccines plus a catch-up campaign for each country. (D) Bar plots of the mean reduction and 95% prediction interval of multidrug resistance for each country.

Across countries, approximately two-thirds of cases, deaths, and DALYs associated with both FQNS and multidrug resistance can be averted through TCV introduction on average, with country-specific ranges from approximately 50% to 75% (figure 2). Over 90% of the total avertable cases of antimicrobial-resistant typhoid fever occurred in south Asia (60%) and sub-Saharan Africa (33%; table 3). Because resistant cases contribute proportionally more to deaths and DALYs, vaccination roll-out was predicted to avert relatively more deaths and DALYs in countries with high prevalence of antimicrobial resistance (table 3). For example, vaccination was predicted to avert 56·9% (95% PI 35–74) of cases and 61·3% (95% PI 36–75) of DALYs in Pakistan (table 3).

## Discussion

The emergence and continued high prevalence of antimicrobial resistance threatens to reverse recent gains in combatting the morbidity and mortality caused by typhoid fever. Vaccination is an important tool—alongside antibiotic stewardship, improvements in diagnostic testing, and drug development—in mitigating the threat posed by drug-resistant pathogens. WHO's Strategic Advisory Group of Experts recommended the routine use of TCVs in typhoid-endemic countries, particularly those with a high prevalence of antimicrobial resistance.^13^ We found that TCV introduction could avert approximately 53·5 million cases of antimicrobial-resistant typhoid fever, with substantial reductions in FQNS in south Asia and multidrug resistance in sub-Saharan Africa.

The predicted number of deaths and DALYs averted by TCV introduction was proportionally greater than the number of cases averted, particularly in countries with high prevalence of antimicrobial resistance. Patients infected with antimicrobial-resistant strains of typhoid fever are likely to have poorer outcomes, including prolonged illness and higher risk of relapse, complications, and possibly death.^21^, ^22^ However, there is also greater uncertainty in the number of deaths and DALYs that can potentially be averted through TCV introduction. More research is needed to better understand the risk of hospitalisation and death from typhoid fever and how it varies for antimicrobial-resistant versus antimicrobial-sensitive strains.

Compared with antimicrobial-sensitive typhoid fever cases, we found that vaccination was predicted to have a greater effect on antimicrobial-resistant typhoid fever cases. The relative proportion of cases that are resistant was predicted to decline by approximately 16% following TCV introduction; however, this varied depending on how recent resistance was assumed to emerge, the typhoid transmission rate (as quantified by R_0_), and the proportion of cases that are symptomatic, which affects treatment-seeking behaviour. These findings are in contrast with an earlier modelling study, which found no effect of vaccination on the proportion of typhoid cases that are resistant.^16^ However, the previous study only considered routine use of TCV (without a catch-up campaign), and the model did not include age structure. Moreover, the previous study did not consider the relatively recent emergence of antimicrobial resistance. These are key factors affecting the proportion of chronic carriers of typhoid who harbour resistant strains as predicted by the models. The development of chronic carriage is more likely among older individuals infected with typhoid. If antimicrobial resistance has emerged relatively recently (within the past 10–20 years), then it is more likely that chronic carriers will be infected with sensitive strains of *S* Typhi, consistent with observations from patients undergoing cholecystectomies in Nepal.^23^ Vaccination with TCVs is predicted to reduce the prevalence of chronic carriage by preventing new carriers, but existing chronic carriers are likely to serve an increasingly important role in contributing to ongoing transmission in the population.^19^ Since the prevalence of resistance is lower among chronic carriers (many of whom were infected before resistance emerged), prevalence of resistance is predicted to decline following vaccine introduction.

TCV roll-out could be particularly urgent in places where resistance has emerged and is spreading rapidly. The most extreme example is Pakistan, where an outbreak of extensively drug-resistant typhoid has been ongoing since 2016.^7^, ^8^, ^9^ The outbreak is widespread, with over 21 000 cases as of mid-2020, over 70% of which were identified as extensively drug resistant; additional mutations might be emerging.^24^ In response, a mass vaccination campaign of children aged 6 months to 10 years was carried out in Hyderabad, Pakistan, in early 2018.^25^ With support from Gavi, in 2019, Pakistan introduced routine immunisation with TCV at age 9 months across the country, along with catch-up campaigns among children aged 6 months to 15 years in the Sindh and Punjab provinces. Our results suggest that TCV introduction can potentially avert up to 75% of antimicrobial-resistant typhoid cases in Pakistan, and is expected to have an even greater effect on deaths and DALYs caused by typhoid fever.

This study has several limitations. Data on typhoid incidence, case fatality risk, and projected vaccination coverage are limited by availability and based on model estimates or approximations for countries where country-specific data were not available. Similarly, prevalence estimates of antimicrobial resistance could be affected by sampling biases.^5^ For some countries and regions, the most recent estimates available were from 10 or more years ago and might not reflect current resistance prevalence. We did not account for trends in antimicrobial resistance over time. Data on prevalence of antimicrobial resistance within and between countries exhibit high heterogeneity,^5^ leading to wide confidence intervals in our analysis. Furthermore, we did not model changes to the prevalence of resistance after vaccination for each country individually; instead, we predicted the change in relative prevalence of antimicrobial resistance for a range of parameter values, and applied those results to the projected incidence values. We also did not consider differences in the molecular mechanisms of drug resistance—for instance, FQNS is often associated with single nucleotide polymorphisms in the *S* Typhi chromosome, whereas multidrug resistance genes are typically carried on plasmids.^5^ Thus, it might be easier for *S* Typhi strains with multidrug resistance to revert to sensitive through plasmid transfer, which we do not account for in our model. Lastly, from a broader perspective, we focused only on routine immunisation with TCV including a catch-up campaign up to age 15 years; we did not consider improvements in sanitation that might happen concurrently, and we did not model paratyphoid, which is not prevented by TCVs but is also responsible for varying proportions of enteric fever.

By integrating recent estimates of typhoid fever incidence and prevalence of antimicrobial resistance with model predictions of the effect of vaccination, we provide a comprehensive analysis of how TCV introduction might reduce the burden of typhoid fever exhibiting FQNS and multidrug resistance. Our study builds on previous work highlighting the cost effectiveness of TCV introduction^3^ by further highlighting how vaccination has the potential to avert a substantial proportion of antimicrobial-resistant typhoid fever, which is responsible for a disproportionate amount of deaths and DALYs caused by typhoid. We highlight the benefits of TCV introduction and underscore the importance of speedy roll-out, particularly in countries with high, increasing, or recently emerging resistance.

## Data sharing

All the underlying data and model code used for the analysis is available from https://github.com/vepitzer/typhoidAMR.

## Declaration of interests

RB is now employed by Merck Sharp & Dohme. AJP has received grants from National Institute for Health Research, the Bill & Melinda Gates Foundation, Wellcome Trust, and Astra Zeneca outside of the submitted work; chairs the UK Department of Health's Joint Committee on Vaccination and Immunisation; and is a member of WHO's Strategic Advisory Group of Experts. VEP has received reimbursement from Merck and Pfizer for travel expenses to Scientific Input Engagements unrelated to the subject of this Article; and is a member of the WHO's Immunization and Vaccine-related Implementation Research Advisory Committee. The views expressed in this manuscript are those of the authors and do not necessarily reflect the views of the Joint Committee on Vaccination and Immunisation, Department of Health, or WHO. All other authors declare no competing interests.
