# Supplementary material for: Estimating the effect of vaccination on antimicrobial-resistant typhoid fever in 73 countries supported by Gavi: a mathematical modelling study
Source: Lancet Infect Dis. 2022 May;22(5):679–91. doi: 10.1016/S1473-3099(21)00627-7 (PMC9021026; doi:10.1016/S1473-3099(21)00627-7)
Supplement: Supplementary appendix [file mmc1.pdf]

THE LANCET
Infectious Diseases

Supplementary appendix

This appendix formed part of the original submission and has been peer reviewed. We post it as supplied by the authors.

Supplement to: Birger, R Antillón M, Bilcke J, et al. Estimating the effect of vaccination on antimicrobial-resistant typhoid fever in 73 countries supported by Gavi: a mathematical modelling study. *Lancet Infect Dis* 2022; published online Feb 3. https://doi.org/10.1016/S1473-3099(21)00627-7.

Appendix

Estimating the impact of vaccination on antimicrobial-resistant typhoid fever in Gavi-73 countries: a mathematical modelling study

Ruthie Birger, Marina Antillón, Joke Bilcke, Christiane Dolacek, Gordon Dougan, Andrew J. Pollard, Kathleen M. Neuzil, Isabel Frost, Ramanan Laxminarayan, Virginia E. Pitzer*

*Corresponding author: virginia.pitzer@yale.edu

Table of Contents

1. Transmission model for predicting the impact of vaccination on the overall burden of typhoid fever 2

1.1 Population age distribution of Gavi-73 countries 2

1.2 Estimating the country-specific R_0 and reporting rate 3

1.3 Predicting the impact of vaccination 3

2. Meta-analysis of the prevalence of antimicrobial resistance 5

3. Description of transmission model including drug resistance and age structure 9

3.1 Vaccination 9

3.2 Model Equations 10

4. Impact of vaccination on the overall burden of typhoid fever 12

4.1 Factors correlated with vaccine impact 12

5. Predicted impact of vaccination on the proportion of cases that are drug resistant 13

6. Impact of vaccination on the burden of FQNS and MDR typhoid fever 16

7. Supplementary references 22

1. Transmission model for predicting the impact of vaccination on the overall burden of typhoid fever

The transmission model for the overall burden of typhoid fever, as described in detail in Bilcke et al.,[1] assumes individuals are born at a rate μN, where N is the total population size, into the susceptible state (S) in the lowest age-stratum. Susceptible individuals can become infected and enter the I_M compartment (infectious, with a possibility of symptoms) at a rate λ (also known as the force of infection). The average duration of infectiousness is equal to $1/\delta$, after which an age-specific fraction θ_a of infectious individuals become life-long chronic carriers (C). The remaining 1- θ_a recover and gain temporary immunity to reinfection (R). Individuals lose immunity at a rate ω and enter state S_R, and are again susceptible to subclinical infection (I_Q) occurring at a rate λ. Individuals who are re-infected are assumed to recover and become temporarily immune again (R). The force of infection, λ, depends on the prevalence of symptomatic and subclinical infections (I_M and I_Q, respectively) and chronic carriers (C), for whom we assume the relative infectiousness is reduced by a factor r. We assume transmission is frequency-dependent, and we allow for lower transmission rates among individuals in the two youngest age groups (i.e. the transmission rate is $m_1\beta_0$ for infants 0-<2 years of age, $m_2\beta_0$ for children 2-<5 years of age, and β_0 for all other age groups), as this provides the best fit to available age-specific incidence data. The basic reproductive number (R_0), defined as the expected number of secondary infections produced by an infectious individual in a fully susceptible population, is related to the average transmission rate across all age groups and the relative contribution of chronic carriers according to the formula: $R_0 = \bar{\beta}(1 + r\delta\bar{\theta}/\mu)/(\delta + \mu)$, where $\bar{\beta}$ and $\bar{\theta}$ are the sum of the respective age-specific parameters weighted by the proportion of the population in each age group. All other model parameters are defined in Table S1.

Table S1: Input parameters of the dynamic transmission model. For each parameter, we list its fixed value or estimated mean and uncertainty distribution, and the data/information on which this was based ('source').

Characteristic	Mean value and uncertainty distribution	Source
Demographic parameters		
Crude birth rate (per year) (μ)	0.0151 for "average" age populations<br>0.0235 for "young" populations<br>0.0360 for "very young" populations	See Figure S1; UN World Population Prospects (WPP) database.[2]
Crude death rate (per year) (μ)	Adjusted to maintain a constant population size	Assumption
Fixed disease parameters		
Mean duration of infectiousness ($1/\delta$)	4 weeks	Hornick et al.[3]
Fraction infected who become carriers (θ_a).	0·003 for individuals <25 years old<br>0·021 for individuals ≥25 years old	Ames and Robins[4]
Duration of immunity ($1/\omega$)	104 weeks	Hornick et al.[3]
Disease parameters with a priori distributions		
Relative transmission rate for children 0-2 years old, (m_1)	0·37, Beta(0·79, 1·36)	A random draw from the parameters for the five sites modelled in Antillón et al.[5]
Relative transmission rate for children 2-5 years old (m_2)	0·68, Beta(1·55, 0·72)	
Relative infectiousness of chronic carriers (r)	0·25, Beta(6·34, 19·4)	Fitted to data from a cluster randomised trial of Vi-polysaccharide vaccine, as described in Antillón et al.[5]
Vaccine-related characteristics		
Initial efficacy of TCV (v)	87·5%, Uniform(80%,95%)	Based on Jin et al.[6] and Voysey et al.[7]; see also Pitzer et al.[8] for validation against recent data for Tybar-TCV.[9]
Waning of vaccine-induced immunity (ω_V)	0·0672 per year,<br>Gamma(1·40, 0·0479)	Re-analysis of Vi-rEPA data;[10] see also Pitzer et al.[8] for validation against recent data for Tybar-TCV.[9]

1.1 Population age distribution of Gavi-73 countries

When identifying the parameter values (for R_0 and the proportion symptomatic) that reproduce the incidence and average age of typhoid infection in each country, it is necessary to account for the demographic profile of the population. However, simulating incidence while accounting for country-specific birth rates and age-distributions in each of the 73 original Gavi-eligible countries would require a massive computational effort. We simplified the process by grouping countries into three standard demographic profiles, selected according to estimates for all low-income countries, lower-middle-income countries, and upper- middle-income countries as listed in the UN World Population Prospects (WPP) database.[2] We refer to these sets of estimates as "very young", "young", and "average" populations, respectively, and assigned each country to a standard demographic profile according to the proportion

of the population under 5 that most closely matched the population <5 year of age in each standard demographic profile: "very young" (16%), "young" (10%), and "average" (7%) (Fig. S1).

1.2 Estimating the country-specific R_0 and reporting rate

For each standard demographic profile, we simulated typhoid incidence and the average age of infection for a wide range of R_0 values. We then used cubic interpolators to approximate the relationship between R_0 and the average age of infection (interpolator #1) and the typhoid incidence rate (interpolator #2). The value of R_0 for each country was estimated by sampling uniformly from uncertainty in the average age of infection (based on the 2017 Global Burden of Disease (GBD)[11] and Antillón et al "Yale burden model" estimates[12]) and matching to the value of R_0 that most closely reproduced the average age of infection given the country's standard demographic profile. Based on interpolator #2, we then calculated the "reporting rate" (i.e. proportion of first infections that are symptomatic, s) by comparing the model-simulated incidence of infection for the corresponding value of R_0 to the estimated incidence of typhoid fever from the GBD and Yale burden models (again sampling uniformly from the uncertainty in the mean typhoid incidence for each country), such that s = (typhoid fever incidence per year according to the burden estimate)/(transmission-model-predicted incidence of infection per year|R_0). Finally, we back-calculated the value of β_0 given the estimated value of R_0 and random samples from the distributions for m_1, m_2, and r. For more details, see Bilcke et al.[1]

1.3 Predicting the impact of vaccination

To simulate the impact of vaccination, we differentiate between immunologically naïve individuals (i.e. who had not been previously infected with typhoid) who successfully mount a protective response to vaccination (V_1) with probability v and those who are already partially immune to clinical infection and successfully immunized (V_2). We assume the former are protected from clinical disease, while the latter are protected from subclinical infection and hence transmission. We assume vaccine-induced immunity wanes exponentially with rate parameter ω_v equal to the inverse of the mean duration of vaccine-induced immunity, which was estimated based on data on vaccine effectiveness over 1-4 years of follow-up for the Vi-rEPA vaccine,[10] as detailed in Antillón et al;[5] this is currently the only available long-term effectiveness data for a TCV. We validated our assumptions about the initial vaccine efficacy (v) and the duration of vaccine-induced immunity by comparing to more recent data for the Tybar-TCV over 2 years of follow-up.[8,9] Vaccine coverage estimates for each country were based on Gavi's demand forecast for 2019-2030 given "unconstrained" (i.e. unlimited) supplies of TCV.[1] The model was simulated for 500 years to reach equilibrium (burn-in period) before introducing vaccination and evaluating the impact over a 10-year time horizon. We ran the model 2000 times for each country, sampling from the distributions of the estimated disease and vaccination parameters (Table S1).

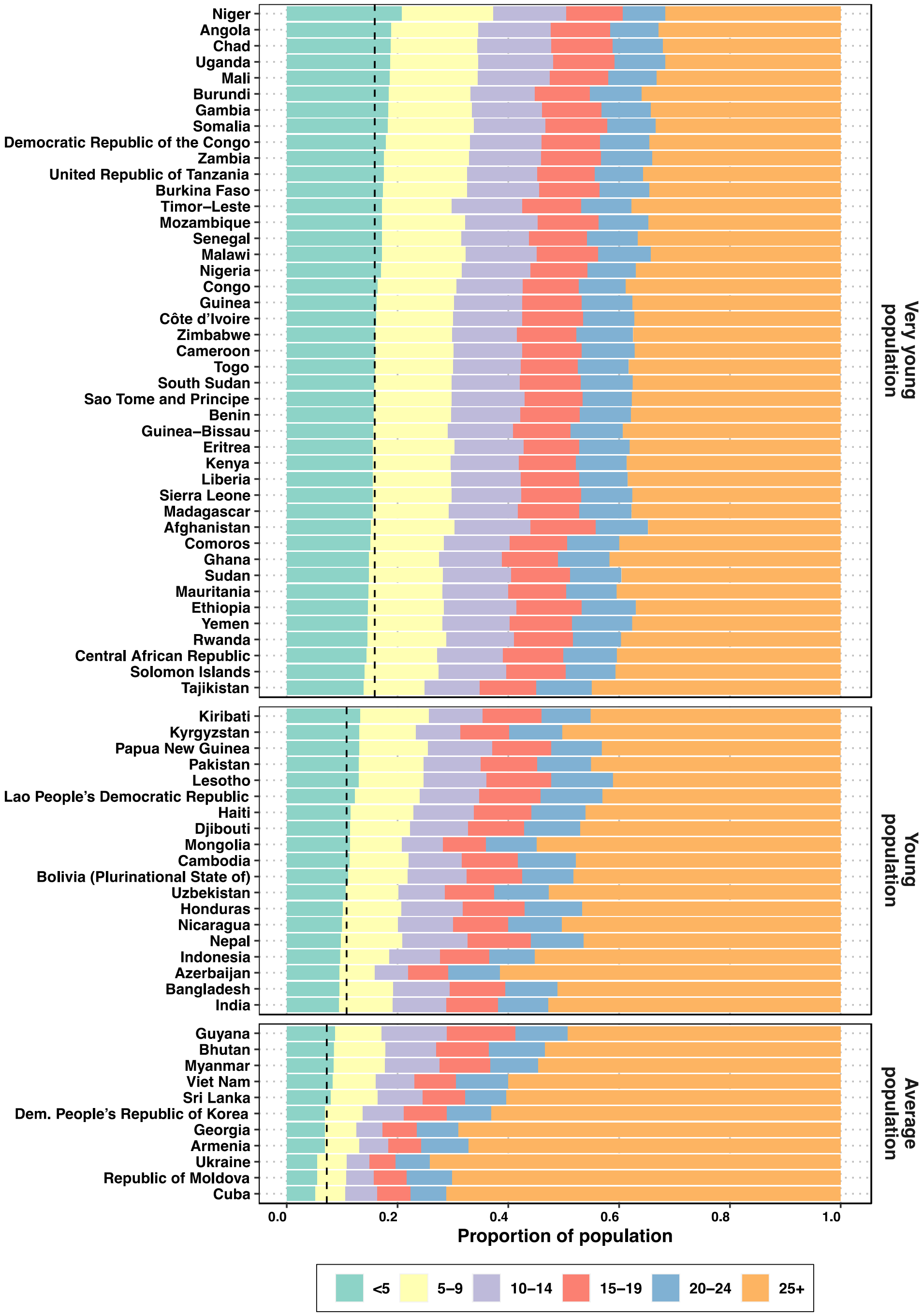

Figure S1. Population age distribution of Gavi-73 countries. The proportion of the population <5 years of age (green), 5-9 years old (yellow), 10-14 years old (purple), 15-19 years old (red), 20-24 years old (blue), and 25 years and older (orange) is plotted for Gavi-73 countries on the horizontal axis. Countries are listed in order from the greatest proportion of the population <5 years old (top) to the lowest proportion <5 years old (bottom) and classified into "very young", "young", and "average" populations (black boxes) based on the closest match to the average under 5 population amongst low-, lower-middle- and upper-middle-income countries, respectively (black dashed lines).

2. *Meta-analysis of the prevalence of antimicrobial resistance*

To estimate the prevalence of fluoroquinolone non-susceptibility (FQNS) and multidrug resistance (MDR) in each country, we used the 'meta' package in R to combine estimates of FQNS and MDR prevalence by country and Global Burden of Disease (GBD) (super-)region, based on data available from two recent systematic reviews.[13,14] We used country-specific estimates of AMR prevalence from all studies conducted since 2010, where available. For countries without published data on AMR prevalence, we used the regional meta-analysis estimate, with the exception of those countries with missing data outlined in the footnotes of Tables S2-S3. We combined regions when a region only had studies from one country, e.g. estimates for Central Africa were derived from all studies in Western and Central Africa. We then used the method of Joseph and Belisle to estimate the parameters of the corresponding beta distributions from the 95% predictions intervals of the FQNS and MDR prevalence estimates (Tables S2-S3).[15]

Table S2. Meta-analysis estimates of fluoroquinolone non-susceptibility for individual countries and regions.

**Super-region, region, or country**	**Source data**	**Estimate from meta-analysis (95% prediction interval)**	**Beta distribution parameters**	
			**alpha**	**beta**
South Asia				
Bangladesh	Studies from the country	0.91 (0.66−0.98)	12.06	1.86
India	Studies from the country	0.90 (0.80−0.95)	55.33	6.85
Nepal	Studies from the country	0.79 (0.64−0.89)	31.49	8.95
Pakistan	Studies from the country	0.75 (0.23−0.97)	2.81	1.42
Others (Bhutan)	South Asia	0.87 (0.79−0.92)	93.33	14.83
Southeast Asia, East Asia, and Oceania				
Cambodia	Studies from the country	0.81 (0.26−0.98)	2.83	1.19
Indonesia	Southeast Asia (only one study from the country)	0.49 (0.04−0.96)	1.13	1.15
All other countries	Southeast Asia	0.49 (0.04−0.96)	1.13	1.15
Sub-Saharan Africa				
Western Sub-Saharan Africa				
Nigeria	Studies from the country	0.01 (0.00−1.00)	0.20	0.36
Burkina Faso	Studies from the country	0.00 (0.00−0.10)	1.00	29.00
Ghana	Western Sub-Saharan Africa (only one study from the country)	0.00 (0.00−1.00)	0.12	0.47
All other countries	Western Sub-Saharan Africa	0.00 (0.00−1.00)	0.12	0.47
Central Sub-Saharan Africa				
DRC	Studies from the country	0.38 (0.31−0.45)	65.93	108.91
All other countries	Western & Central Sub-Saharan Africa combined	0.02 (0.00−0.51)	0.53	1.78
Eastern Sub-Saharan Africa				
Kenya	Eastern Sub-Saharan Africa (only one study from the country)	0.23 (0.16−0.32)	26.53	85.25
Tanzania	Eastern Sub-Saharan Africa (only one study from the country)	0.23 (0.16−0.32)	26.53	85.25
All other countries	Eastern Sub-Saharan Africa	0.23 (0.16−0.32)	26.53	85.25
Southern Sub-Saharan Africa				
All countries	Sub-Saharan Africa	0.04 (0.00−0.35)	0.95	7.91
Central Europe, Eastern Europe, and Central Asia				
Uzbekistan	Older studies (~ 2000s)	0.91 (0.86−0.95)	138.7	13.98
Tajikistan	One older study (~ 1990s)	0.82 (0.65−0.93)	28	6
All countries	Older studies (~ 2000s)	0.90 (0.85−0.93)	166.3	19.75
North Africa and the Middle East				
Iraq	Only study from country and region available	0.06 (0.02−0.14)	4.35	56.72
All other countries	Study from Iraq	0.06 (0.02−0.14)	4.35	56.72
Latin America and the Caribbean				
Peru	Only study from country and region available	0.24 (0.13−0.42)	8.28	23.88
All other countries	Study from Peru	0.24 (0.13−0.42)	8.28	23.88
**If FQNS data was missing:**				

For North Africa & the Middle East and Latin America & the Caribbean, each region had one study of FQNS in the Browne et al, 2020 review; there was no obvious neighboring region with which to combine estimates, so the estimates from the single study were projected to the whole super-region.<br>For Central Europe, Eastern Europe, & Central Asia, data from a multi-country study from 2002-2007 was used.[16]<br>For East Asia, four studies from China published in between 2000-2010 in Browne et al, 2020, were used.<br>For Southern Africa, the meta-analysis estimate from all sub-Saharan African studies was used.

Table S3. Meta-analysis estimates of multidrug resistance for individual countries and regions.

Super-region, region, or country	Source data	Estimate from meta-analysis (95% prediction interval)	Beta distribution parameters	
			alpha	beta
South Asia				
Bangladesh	Studies from the country	0.30 (0.25−0.36)	79.28	184.32
India	Studies from the country	0.02 (0.01−0.05)	6.08	233.75
Nepal	Studies from the country	0.01 (0.00−0.03)	4.26	313.49
Pakistan	Studies from the country	0.50 (0.38−0.61)	33.49	33.72
Others (Bhutan)	South Asia	0.03 (0.02−0.07)	7.64	192.17
Southeast Asia, East Asia, and Oceania				
Cambodia	Studies from the country	0.66 (0.30−0.90)	5.01	3.04
Indonesia	Southeast Asia (only one study from the country)	0.19 (0.00−0.94)	0.64	1.10
All other countries	Southeast Asia	0.19 (0.00−0.94)	0.64	1.10
Sub-Saharan Africa				
Western Sub-Saharan Africa				
Burkina Faso	Studies from the country	0.00 (0.00−0.10)	1.00	29.00
Nigeria	Western Sub-Saharan Africa (only one study in the country)	0.00 (0.00−1.00)	0.11	0.15
Ghana	Western Sub-Saharan Africa (only one study in the country)	0.00 (0.00−1.00)	0.11	0.15
All other countries	Western Sub-Saharan Africa (only one study in the country)	0.00 (0.00−1.00)	0.11	0.15
Central Sub-Saharan Africa				
DRC	Studies from the country	0.36 (0.30−0.43)	69.77	122.32
All other countries	Western & Central Sub-Saharan Africa combined	0.29 (0.02−0.88)	1.11	1.78
Eastern Sub-Saharan Africa				
Kenya	Eastern Sub-Saharan Africa (only one study in the country)	0.59 (0.36−0.79)	10.30	7.38
Tanzania	Eastern Sub-Saharan Africa (only one study in the country)	0.59 (0.36−0.79)	10.30	7.38
All other countries	Eastern Sub-Saharan Africa	0.59 (0.36−0.79)	10.30	7.38
Southern Sub-Saharan Africa				
All countries	Sub-Saharan Africa	0.38 (0.08−0.80)	2.16	3.06
Central Europe, Eastern Europe, and Central Asia				
Uzbekistan	Older studies (~ 2000s)	0.13 (0.09−0.20)	21.69	134.8
Tajikistan	One older study (~ 1990s)	0.91 (0.76−0.98)	31.00	3.00
All countries	Older studies (~ 2000s)	0.38 (0.06−0.86)	1.68	2.26
North Africa and the Middle East				
All countries	Older studies (~ 2000s)	0.07 (0.02−0.24)	2.356	21.57
Latin America and the Caribbean				
All countries	Americas	0.02 (0.00−0.15)	1.17	23.01
**If MDR data was missing:**<br>For North Africa & the Middle East, the meta-analysis estimate from studies from North Africa and the Middle East was used, where the midpoint year of data collection fell between 2000-2010 in the Browne et al, 2020 review.[7]<br>For Latin America & the Caribbean, the meta-analysis estimate of North American data from the US[17] and studies from Mexico and Colombia was used.[18,19]<br>For Central Europe, Eastern Europe, & Central Asia, data from a multi-country study from 2002-2007 was used.[16]<br>For East Asia, a single study from China was used.[20]<br>For Southern Africa, the meta-analysis estimate from all sub-Saharan African studies was used.				

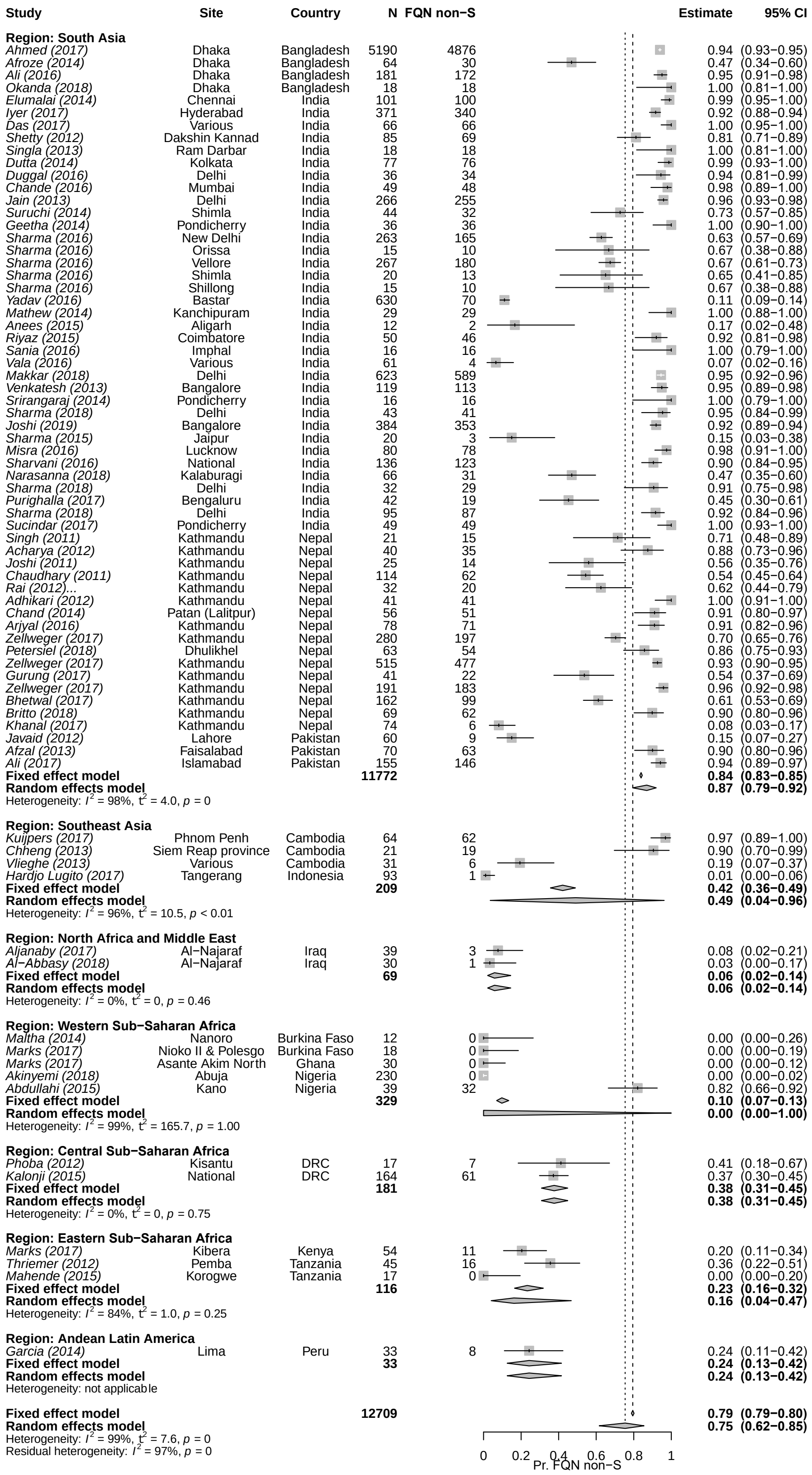

Figure S2. Studies contributing to meta-analysis of prevalence of fluoroquinolone non-susceptibility. Studies are grouped by GBD region. Fixed and random effects model estimates are shown for each region, along with measures of heterogeneity. For regions or super-regions without data, our assumptions are outlined in Table S1.

Study	Site	Country	N	MDR	Estimate	95% CI
**Region: South Asia**						
Leung (2012)	Dhaka	Bangladesh	207	71	0.34	(0.28−0.41)
Afroze (2014)	Dhaka	Bangladesh	71	23	0.32	(0.22−0.45)
Yu (2017)	Dhaka	Bangladesh	1425	371	0.26	(0.24−0.28)
Okanda (2018)	Dhaka	Bangladesh	18	7	0.39	(0.17−0.64)
Elumalai (2014)	Chennai	India	101	5	0.05	(0.02−0.11)
Iyer (2017)	Hyderabad	India	408	4	0.01	(0.00−0.02)
Das (2017)	Various	India	66	13	0.20	(0.11−0.31)
Shetty (2012)	Dakshin Kannad	India	85	2	0.02	(0.00−0.08)
Dutta (2014)	Kolkata	India	77	14	0.18	(0.10−0.29)
Duggal (2016)	Delhi	India	36	2	0.06	(0.01−0.19)
Chande (2016)	Mumbai	India	108	0	0.00	(0.00−0.03)
Jain (2013)	Delhi	India	266	9	0.03	(0.02−0.06)
Suruchi (2014)	Shimla	India	51	0	0.00	(0.00−0.07)
Geetha (2014)	Pondicherry	India	36	2	0.06	(0.01−0.19)
Sharma (2016)	Orissa	India	15	0	0.00	(0.00−0.22)
Sharma (2016)	Shillong	India	15	0	0.00	(0.00−0.22)
Sharma (2016)	Shimla	India	20	0	0.00	(0.00−0.17)
Yadav (2016)	Bastar	India	630	130	0.21	(0.18−0.24)
Anees (2015)	Aligarh	India	12	8	0.67	(0.35−0.90)
Riyaz (2015)	Coimbatore	India	50	2	0.04	(0.00−0.14)
Sania (2016)	Imphal	India	16	0	0.00	(0.00−0.21)
Makkar (2018)	Delhi	India	623	16	0.03	(0.01−0.04)
Venkatesh (2013)	Bangalore	India	251	0	0.00	(0.00−0.01)
Srirangaraj (2014)	Pondicherry	India	16	1	0.06	(0.00−0.30)
Joshi (2019)	Bangalore	India	384	6	0.02	(0.01−0.03)
Ramesh (2016)	East	India	10	0	0.00	(0.00−0.31)
Misra (2016)	Lucknow	India	80	5	0.06	(0.02−0.14)
Sharvani (2016)	National	India	167	3	0.02	(0.00−0.05)
Narasanna (2018)	Kalaburagi	India	66	0	0.00	(0.00−0.05)
Purighalla (2017)	Bengaluru	India	42	0	0.00	(0.00−0.08)
Wankhede (2018)	Pune	India	27	0	0.00	(0.00−0.13)
Koirala (2013)	Patan (Lalitpur)	Nepal	132	0	0.00	(0.00−0.03)
Raza (2012)	Kathmandu	Nepal	47	3	0.06	(0.01−0.18)
Zellweger (2017)	Kathmandu	Nepal	564	1	0.00	(0.00−0.01)
Chand (2014)	Patan (Lalitpur)	Nepal	56	0	0.00	(0.00−0.06)
Zellweger (2017)	Kathmandu	Nepal	406	4	0.01	(0.00−0.03)
Zellweger (2017)	Kathmandu	Nepal	281	23	0.08	(0.05−0.12)
Zellweger (2017)	Kathmandu	Nepal	495	4	0.01	(0.00−0.02)
Zellweger (2017)	Kathmandu	Nepal	187	4	0.02	(0.01−0.05)
Britto (2018)	Kathmandu	Nepal	69	2	0.03	(0.00−0.10)
Khanal (2017)	Kathmandu	Nepal	74	0	0.00	(0.00−0.05)
Qamar (2014)	National	Pakistan	2552	1671	0.65	(0.64−0.67)
Afzal (2013)	Faisalabad	Pakistan	80	21	0.26	(0.17−0.37)
Ali (2017)	Islamabad	Pakistan	155	69	0.45	(0.37−0.53)
Qamar (2018)	Karachi & Hyderabad	Pakistan	1961	1035	0.53	(0.51−0.55)
Zehra (2017)	Karachi	Pakistan	270	154	0.57	(0.51−0.63)
**Fixed effect model**			**12708**		**0.29**	**(0.28−0.30)**
**Random effects model**					**0.03**	**(0.02−0.07)**
Heterogeneity: I^2 = 99%, τ^2 = 4.9, p = 0						
**Region: Central Sub−Saharan Africa**						
Phoba (2012)	Kisantu	DRC	17	4	0.24	(0.07−0.50)
Phoba (2014)	Sud−Ubangi	DRC	18	6	0.33	(0.13−0.59)
Kalonji (2015)	National	DRC	164	62	0.38	(0.30−0.46)
**Fixed effect model**			**199**		**0.36**	**(0.30−0.43)**
**Random effects model**					**0.36**	**(0.30−0.43)**
Heterogeneity: I^2 = 0%, τ^2 = 0, p = 0.50						
**Region: Eastern Sub−Saharan Africa**						
Marks (2017)	Kibera	Kenya	54	40	0.74	(0.60−0.85)
Thriemer (2012)	Pemba	Tanzania	45	19	0.42	(0.28−0.58)
**Fixed effect model**			**99**		**0.60**	**(0.50−0.69)**
**Random effects model**					**0.59**	**(0.36−0.79)**
Heterogeneity: I^2 = 80%, τ^2 = 0.4, p < 0.01						
**Region: Southeast Asia**						
Kuijpers (2017)	Phnom Penh	Cambodia	64	27	0.42	(0.30−0.55)
Chheng (2013)	Siem Reap province	Cambodia	21	18	0.86	(0.64−0.97)
Lugito (2017)	Tangerang	Indonesia	46	0	0.00	(0.00−0.08)
**Fixed effect model**			**131**		**0.34**	**(0.27−0.43)**
**Random effects model**					**0.19**	**(0.00−0.94)**
Heterogeneity: I^2 = 97%, τ^2 = 11.8, p < 0.01						
**Region: Western Sub−Saharan Africa**						
Maltha (2014)	Nanoro	Burkina Faso	12	0	0.00	(0.00−0.26)
Marks (2017)	Nioko II & Polesgo	Burkina Faso	18	0	0.00	(0.00−0.19)
Marks (2017)	Asante Akim North	Ghana	30	19	0.63	(0.44−0.80)
Akinyemi (2018)	Lagos	Nigeria	58	58	1.00	(0.94−1.00)
**Fixed effect model**			**118**		**0.65**	**(0.56−0.73)**
**Random effects model**					**0.00**	**(0.00−1.00)**
Heterogeneity: I^2 = 99%, τ^2 = 203.5, p = 1.00						
**Fixed effect model**			**13255**		**0.30**	**(0.29−0.30)**
**Random effects model**					**0.05**	**(0.02−0.11)**
Heterogeneity: I^2 = 99%, τ^2 = 7.2, p = 0						
Residual heterogeneity: I^2 = 97%, p = 0						

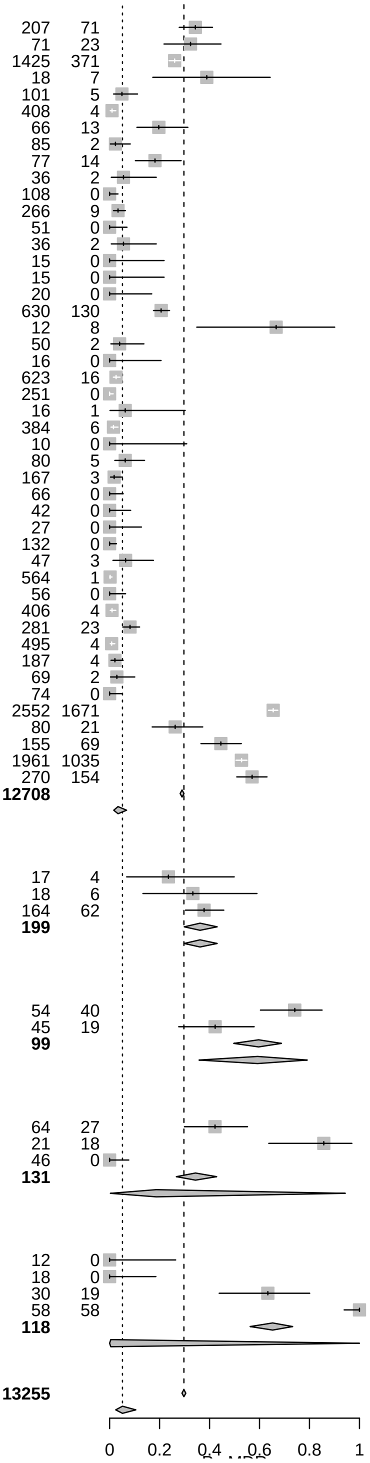

Figure S3. Studies contributing to meta-analysis of prevalence of multidrug resistance. Studies are grouped by GBD region. Fixed and random effects model estimates are shown for each region, along with measures of heterogeneity. For regions or super-regions without data, our assumptions are outlined in Table S2.

3. *Description of transmission model including drug resistance and age structure*

We assumed individuals are born into the fully susceptible (S_1) compartment in the youngest age group at per capita birth rate b multiplied by the total population *N*. Fully susceptible individuals can be infected with either drug-sensitive or drug-resistant typhoid with force of infection λ_S or λ_R, respectively. When infected with drug-sensitive typhoid, individuals enter either the primary infection compartment, I_S for those who do not get treated, or I_T for those who do (at rate $\sigma \times \tau$, representing the proportion symptomatic times proportion treated). Treated, drug-sensitive individuals recover at rate γ, but also develop drug resistance at rate ρ. Untreated drug-sensitive individuals stay infected for duration $1/\delta_{1S}$, after which a proportion (θ_{1S}) become chronic carriers (C_S), and the rest recover ($1-\theta_{1S}-\alpha$) or die from disease (α). Individuals can also be infected with drug-resistant typhoid (I_R) either by acquiring resistance after being treated for drug-sensitive typhoid (ρI_T), or by being directly infected with drug-resistant typhoid ($\lambda_R S$). Drug-resistant infected individuals stay infected for duration $1/\delta_{1R}$, after which a proportion (θ_{1R}) become chronic resistant carriers (C_R), and the rest recover ($1-\theta_{1R}-\alpha$) or die from disease (α). Recovered individuals (R) lose immunity at rate ω_2, and enter a second susceptible category (S_2). Individuals in S_2 can be reinfected with either sensitive or resistant subclinical infection and move into compartments I_{2S} or I_{2R}, respectively. As with primary infection, subclinical infection lasts for a certain duration ($1/\delta_{2S}$, $1/\delta_{2R}$), and then a certain proportion of those infected (θ_{2S}, θ_{2R}) become chronic carriers while the rest recover. The model diagram is included as Figure S3. Additionally, all individuals experience natural mortality at death rate μ, and age into the next age group at a rate reciprocal to duration of time spent in the age group (e.g. 1/3 years^{-1} for age group 2-5 years). We have excluded age structure from the model equations below for simplicity, but the equations are identical for each age group except for the birth rate and age-specific vaccination coverage.

The forces of infection λ_S and λ_R for each age group are calculated as follows, where r_c and r_R are the relative risks for transmission from chronic carriers and those infected with drug-resistant typhoid, respectively:

$$\lambda_{S_a} = \beta_a \sum_{\text{age groups}} (I_{1S} + I_{2S} + r_c C_S)$$
$$\lambda_{R_a} = r_R \beta_a \sum_{\text{age groups}} (I_{1R} + I_{2R} + r_c C_R)$$

The effective contact rate, β_a, is age-specific; we assume the youngest age groups (infants 0 to <2 years of age and children 2 to <5 years of age) have a lower risk of infection with *S.* Typhi, represented by coefficients m_1 and m_2, respectively, consistent with best-fit models for age-specific incidence data. These coefficients are applied to β as calculated from R_0:[1,21]

$$\beta = \frac{R_0(b+\delta)}{\left(1+\frac{\delta\theta\, r_C}{b}\right)}$$

The estimated model parameters are listed in Table 1 of the main text, while the fixed model parameters are described in Table S3. We ran the model for a burn-in period of 200 years to reach quasi-equilibrium and introduced resistance 10 years before vaccination by setting the resistance parameters ($\rho, r_R, \delta_{1R}, \delta_{2R}$) to non-zero values after that timepoint; in sensitivity analyses, we examined introducing resistance 5 or 20 years before vaccination.

3.1 Vaccination

When vaccination is rolled out, individuals enter the vaccinated compartment (V_1) from S_1 at rate $e \times \kappa$, representing initial vaccine efficacy times vaccination coverage (i.e. assuming all-or-nothing protection). Individuals leave the vaccinated compartment at rate ω_1 representing waning vaccine-induced immunity, and return to the fully-susceptible compartment (S_1). Individuals in the S_2 and R compartments can also get vaccinated at the same rate; they go into the vaccinated, previously infected compartment (V_2), and when immunity wanes, go back to S_2. Two different scenarios were modeled, one assuming no vaccination and the other assuming routine vaccination at 9 months of age with a catch-up campaign for all individuals up to 15 years of age at the time of rollout. These were based on results from Bilcke et al,[1] where it was shown that when vaccination was cost-effective over no

vaccination, the best strategy was to include a catch-up campaign to 15 years of age as compared to routine vaccination alone or routine vaccination with a campaign up to 5 years of age. Impact was assessed over a 10-year time horizon.

3.2 Model Equations

$$\frac{dS_1}{dt} = bN - (\lambda_S + \lambda_R)S_1 + \omega_1 V + (1 - e\kappa)S_1 - \mu S_1$$

$$\frac{dV_1}{dt} = e\kappa S_1 - \omega_1 V_1 - \mu V_1$$

$$\frac{dV_2}{dt} = e\kappa(R + S_2) - \omega_1 V_2 - \mu V_2$$

$$\frac{dI_{1S}}{dt} = (1 - s\tau)\lambda_S S - \delta_{1S} I_{1S} - \mu I_{1S}$$

$$\frac{dI_T}{dt} = s\tau\lambda_S S - \gamma I_T - \rho I_T - \mu I_T$$

$$\frac{dR}{dt} = \delta_{1S}(1 - \theta_{1S} - \alpha)I_{1S} + \delta_{1R}(1 - \theta_{1R} - \alpha)I_{1R} + \delta_{2S}(1 - \theta_{2S})I_{2S} + \delta_{2R}(1 - \theta_{2R})I_{2R} + \gamma I_T - \omega_2 R - \mu R$$

$$\frac{dI_{1R}}{dt} = \lambda_R S - \delta_{1R} + \rho I_T - \mu I_R$$

$$\frac{dC_S}{dt} = \delta_{1S}\theta_{1S} I_{1S} + \delta_{2S}\theta_{2S} I_{2S} - \mu C_S$$

$$\frac{dC_R}{dt} = \delta_{1R}\theta_{1R} I_{1R} + \delta_{2R}\theta_{2R} I_{2R} - \mu C_R$$

$$\frac{dS_2}{dt} = \omega_2 R + \omega_1 V_2 - (\lambda_S + \lambda_R)S_2 - \mu S_2$$

$$\frac{dI_{2S}}{dt} = \lambda_S S_2 - \delta_{2S} I_{2S} - \mu I_{2S}$$

$$\frac{dI_{2R}}{dt} = \lambda_R S_2 - \delta_{2R} I_{2R} - \mu I_{2R}$$

Table S4. Fixed parameters for the transmission model with drug resistance.

**Parameter Description**	**Parameter name**	**Value**	**Source/Assumptions**
Relative transmission rate for children 0-2 years old, (β_1/β_0)	m_1	0·37	Antillón et al.[5]
Relative transmission rate for children 2-5 years old (β_2/β_0)	m_2	0·68	Antillón et al.[5]
Duration of infectiousness, primary infection, sensitive	$1/d_{1S}$	4 weeks	Hornick et al.[3]
Duration of infectiousness, secondary infection, sensitive	$1/d_{2S}$	4 weeks	Hornick et al.[3]
Proportion who die from disease	α	0·005	Crump et al.,[22] Pieters et al.[23]
Duration of immunity from natural infection	$1/\omega_2$	104 weeks	Hornick et al.[3]
Duration of vaccine-induced immunity	$1/\omega_1$	15 years	Antillón et al.[5]
Birth rate	b	36·6 births per 1000 people per year	Based on the demographic profile of low-middle income population, according to UN World Population Prospects database[2]
Death rate	μ	Adjusted to match birth rate, 0·0695/week	
Vaccine efficacy	e	87·5%	Jin et al.,[6] Voysey et al.[7]

Fraction infected who become carriers, primary infection, sensitive	θ_{1S}	0·003 for ages 0-25, 0·021 for 25+	Ames and Robins[4]
Fraction infected who become carriers, secondary infection, sensitive	θ_{2S}	0·003 for ages 0-25, 0·021 for 25+	Ames and Robins[4]
Fraction infected who become carriers, primary infection, resistant	θ_{1R}	0·003 for ages 0-25, 0·021 for 25+	Ames and Robins[4]
Fraction infected who become carriers, secondary infection, resistant	θ_{2R}	0·003 for ages 0-25, 0·021 for 25+	Ames and Robins[4]
Relative infectiousness of chronic carriers	r_C	0·35	Antillón et al.[5]
Proportion seeking treatment	τ	0·57	Bilcke et al.,[1] Antillón et al.[12]
Recovery rate with treatment	γ	1 week^{-1}	Hornick et al.[3]

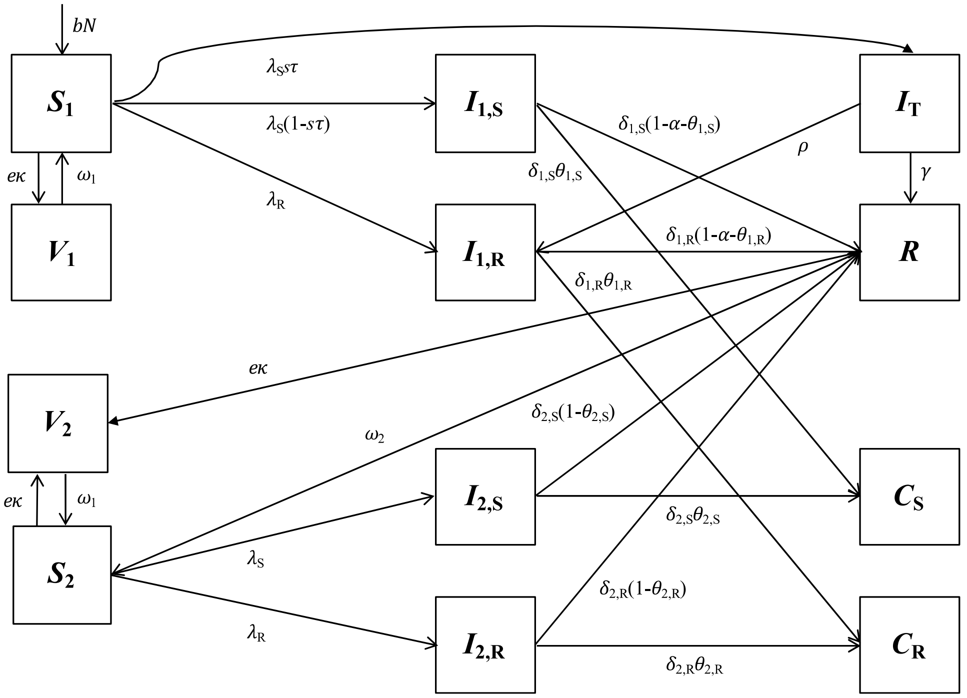

Figure S4. Diagram of the transmission model including drug-resistant states. Model parameters are described in Table S3.

4. Impact of vaccination on the overall burden of typhoid fever

<u>4.1 Factors correlated with vaccine impact</u>

To better understand differences in the predicted impact of introduction of TCV routine immunization plus a catch-up campaign to age 15 years across countries, we plotted the mean percentage of typhoid cases averted versus three country-specific parameters (the estimated routine vaccination coverage, average age of typhoid infections, and the population age distribution) for each of the Gavi-73 countries (Figure S5) and examined the resulting associations.

We tested linear regression models that included vaccination coverage, average age of infection, and population age distribution as a categorical variable. A model including all three variables showed no significant association with the average age of infection. We then tested models including vaccination coverage and population age distribution, with and without an interaction term, and found there was no evidence for an interaction. The best-fit linear regression model with lowest AIC included only vaccine coverage and the population age distribution (Table S5).

Countries in which the age distribution of the population was younger tended to have a greater predicted reduction in typhoid fever incidence ($p<0.0001$) (Table S5, Figure S5A). Within each population age group, countries with a higher expected vaccine coverage also tended to have a greater predicted reduction in typhoid fever incidence (0.3% increase in the percent of cases averted for each 1% increase in coverage, $p<0.0001$) (Table S5, Figure S5B).

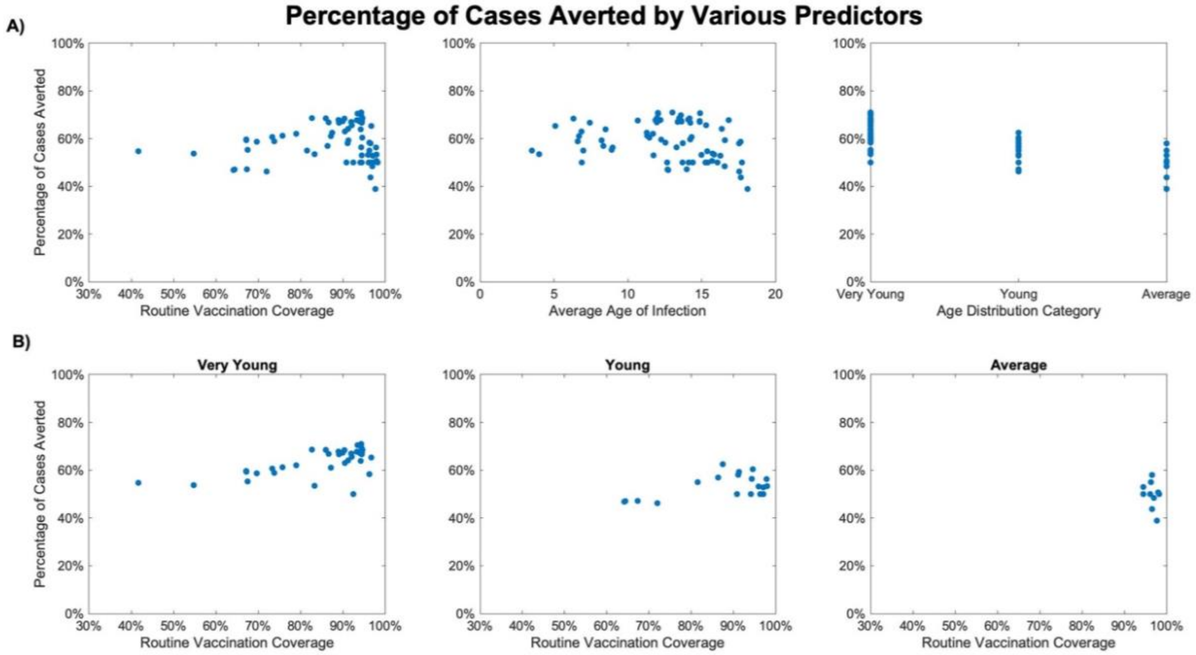

Figure S5. Factors associated with variation in TCV impact across Gavi-73 countries. (A) The mean percentage of typhoid cases predicted to be averted through introduction of routine immunization with TCV plus a catch-up campaign to 15 years of age is plotted against the forecasted routine vaccination coverage, average age of typhoid infections, and population age distribution for each country. (B) For each population age distribution, we plotted the percentage of cases averted versus the estimated routine vaccination coverage.

Table S5. Factors associated with variation in TCV impact across Gavi-73 countries.

Variable	Coefficient	SE	t-statistic	p-value
**(Intercept)**	41.353	3.8936	10.621	<0.0001
**Vaccine coverage**	0.2662	0.0448	5.9365	<0.0001
**Age distribution (young vs very young)**	-11.407	1.1834	-9.6397	<0.0001
**Age distribution (average vs very young)**	-17.212	1.5261	-11.279	<0.0001

R-squared: 0.725, AIC: 423.4

5. *Predicted impact of vaccination on the proportion of cases that are drug resistant*

To summarize the impact of vaccination on the prevalence of drug resistance, we estimated the mean of the proportional difference between the TCV versus no vaccination scenario over the 10 years for each parameter set using the following equation:

$$\Delta_R = \frac{1}{10}\sum_{y=1}^{10} \frac{p_{R,y_{NoVacc}} - p_{R,y_{Camp15}}}{p_{R,y_{NoVacc}}}.$$

where Δ_R is the change in the relative prevalence of drug resistance, $p_{R,y_{NoVacc}}$ is the proportion of incident cases that are drug resistant in year y under the no vaccination scenario and $p_{R,y_{Camp15}}$ is the proportion of incident cases that are drug resistant in year y after vaccine introduction for the routine vaccination plus catch-up campaign scenario (Figure S6).

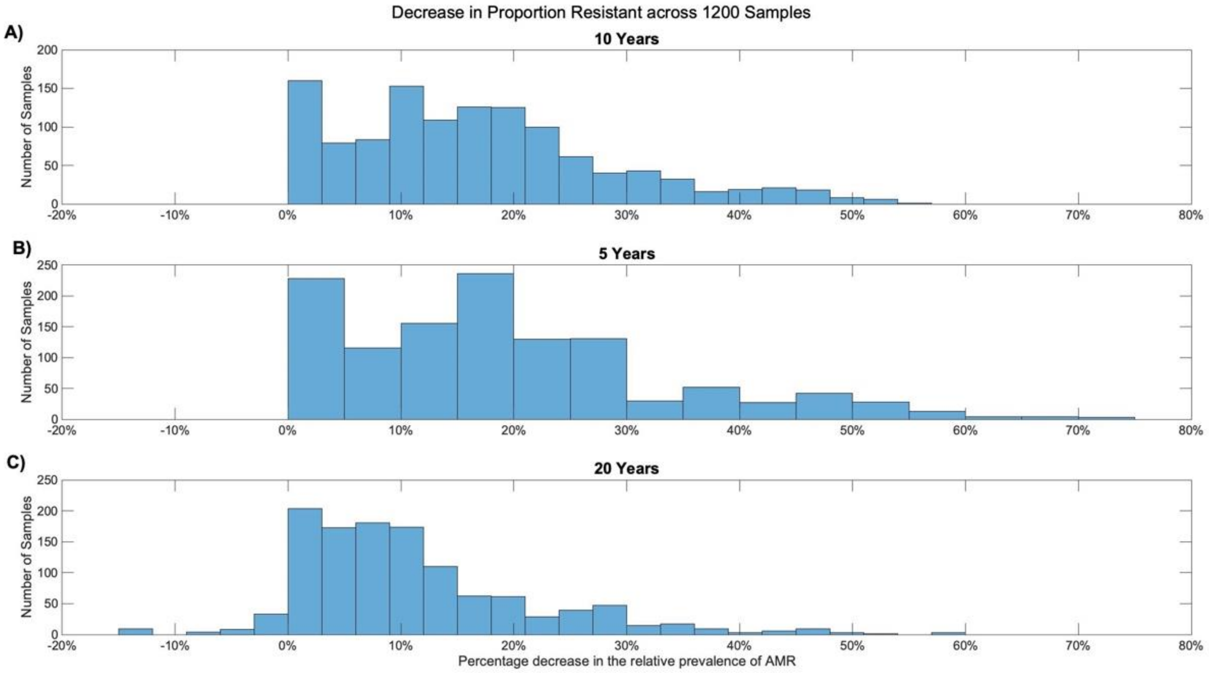

Figure S6. Predicted impact of vaccination on the relative prevalence of antimicrobial resistance. Histograms of the model-predicted decrease in the relative prevalence of AMR over 10 years following TCV introduction are shown, assuming resistance emerges (A) 10 years prior to vaccine introduction (base-case scenario), (B) 20 years prior to vaccine introduction, and (C) 5 years prior to vaccine introduction. Results are based on 1200 samples of the parameter values outlined in Table 1 of the main text.

We tested the (linear) correlations between the predicted decrease in the relative prevalence of AMR and parameters for which we had country-specific data (R_0, the proportion symptomatic, and vaccine coverage) as well as the parameters governing initial resistance prevalence (rate of resistance acquisition, rate of recovery from primary resistant infection, rate of recovery from subclinical resistant infection, and relative risk of transmission of the resistant strain) for the base-case scenario. We found very weak to moderate correlations between these parameters and predicted decrease in the relative prevalence of AMR (Figure S7). There was no clear pattern between the initial prevalence of resistance (which is governed by the resistance parameters) and the change in the relative prevalence of AMR following TCV introduction, although there tended to be slightly smaller reductions in the prevalence of AMR when the initial prevalence was predicted to be very high or very low.

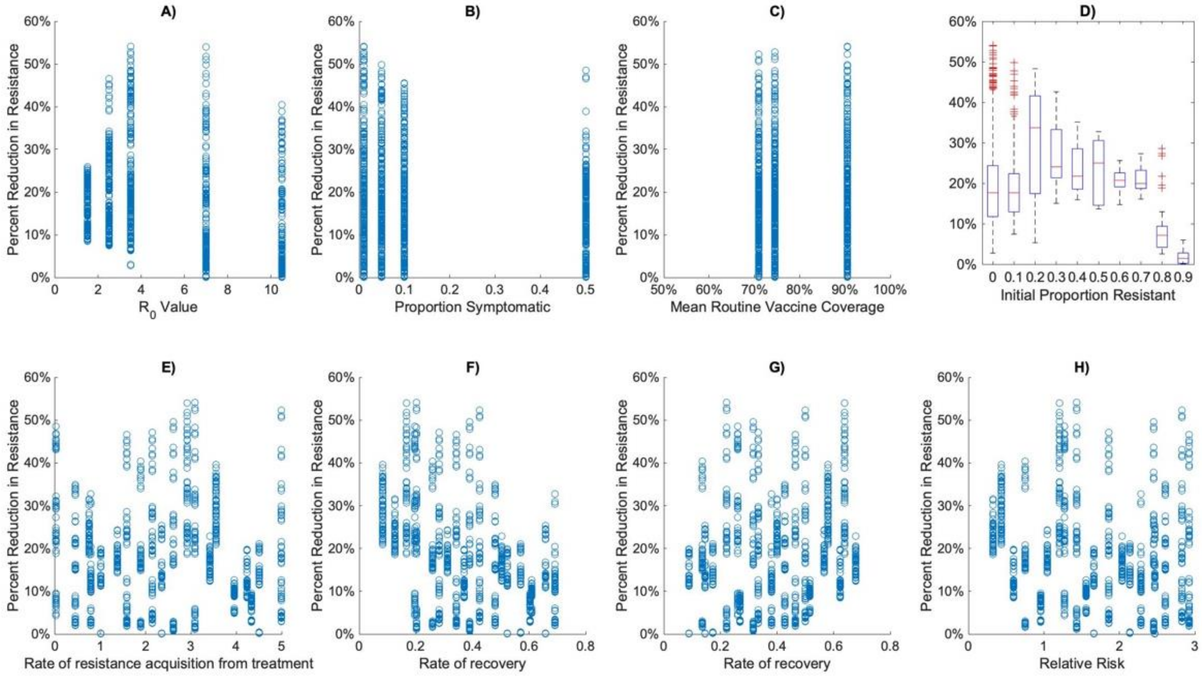

Figure S7. Relationships between model parameters and relative prevalence of AMR after TCV roll-out. Scatterplots show the relationship between each parameter sampled and the range of 1200 model-predicted differences in proportion of cases resistant 10 years after TCV roll-out. Panels A-C show the relationship between the percent reduction in the proportion resistant and the parameters for which there is country-specific data: (A) R_0, corr = -0.32; (B) proportion symptomatic, corr = -0.14; (C) mean vaccine coverage over 10 years, corr = 0.05. Panel (D) shows boxplots of the change in proportion resistant across the generated initial resistance values. Panels (E-H) show the correlations between the parameters governing the initial resistance proportion and change in the relative prevalence of AMR: (E) rate of resistance acquisition, corr =-0.10; (F) rate of recovery from primary resistant infection, corr = -0.51; (G) rate of recovery from subclinical resistant infection, corr = 0.24; (H) relative risk of transmission of resistant infection, corr = -0.20.

To evaluate the influence of country-specific parameters on the predicted decrease in the relative prevalence of AMR, we used linear regression to model the association between the relative change in the proportion resistant (outcome variable) and model parameters that varied across countries (R_0, the proportion of infections symptomatic, and the vaccine coverage). We tested a linear regression model that included R_0, the proportion symptomatic, and vaccine coverage. There was no evidence for vaccine coverage being a predictor, so we tested a model that included R_0 and the proportion symptomatic, which showed evidence of both factors being predictors and had the lowest AIC (Table S6).

Table S6. Factors affecting the model-predicted change in the proportion resistant.

Variable	Coefficient	SE	t-statistic	p-value
**(Intercept)**	23.786	0.63214	37.628	<0.0001
**R_0**	-1.138	0.095523	-11.914	<0.0001
**Proportion symptomatic**	-8.3048	1.6162	-5.1385	<0.0001

R-squared: 0.123, AIC: 9157

The change in the relative prevalence of AMR for each country was then sampled based on the estimated relationship for the country-specific model parameters. We plotted the sampled values of the change in relative prevalence of AMR for two example countries—one with a low estimated R_0 (Somalia) and one with a high

estimated R_0 (Bhutan)—to compare, and found only minor differences in the predicted change in the relative prevalence of AMR across the two countries (Figure S8).

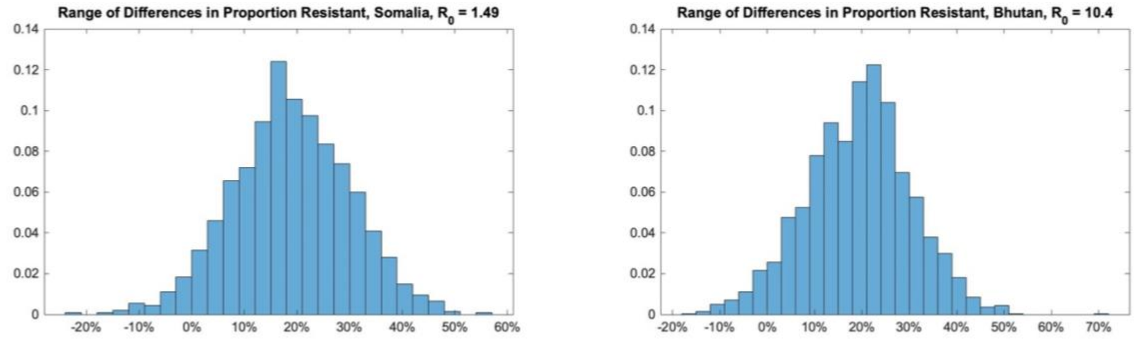

Figure S8. Model-predicted change in proportion resistant for two example countries. Histograms of the model-predicted decrease in the relative prevalence of AMR over 10 years following TCV introduction using the regression model in Table S6 applied to Somalia (low R_0) and Bhutan (high R_0).

We also examined the relative contributions of transmitted versus de novo resistance to the overall proportion of resistant cases before and after vaccine introduction predicted by the model with drug resistance. The mean proportion of cases due to transmitted resistance declined slightly (from approximately 18% to approximately 14%) with TCV roll-out (Figure S9). However, there were very large uncertainty bounds, ranging from 0 to 100%.

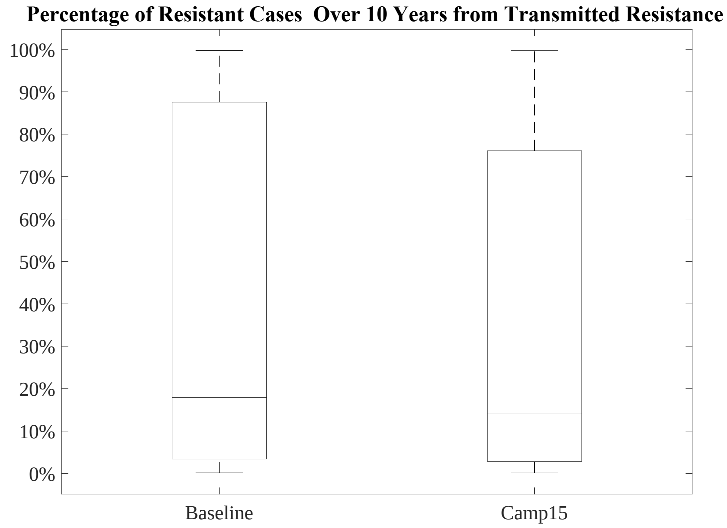

Figure S9. Proportion of resistance due to transmitted vs de novo resistance with and without TCV roll-out. Boxes represent the lower and upper quartiles of the predicted percentage of resistance associated with transmission, with the line in the middle representing the median prediction and dashed whiskers representing the range of model predictions.

6. Impact of vaccination on the burden of FQNS and MDR typhoid fever

Table S7. Baseline burden and vaccine impact on FQNS typhoid fever. Results are for a 10-year time horizon. Countries are grouped by GBD super-region.

Country	Baseline FQNS cases (thousands) (95% prediction interval)	Baseline FQNS deaths (95% prediction interval)	Baseline FQNS DALYs (thousands) (95% prediction interval)	FQNS cases averted (thousands) (95% prediction interval)	FQNS deaths averted (95% prediction interval)	FQNS DALYs averted (thousands) (95% prediction interval)
Sub-Saharan Africa						
**Angola**	148 (0.098-693)	560 (0-25,244)	22 (0.013-971)	97 (0.068-506)	382 (0-15,464)	16 (0.005-611)
**Benin**	139 (0.627-1,319)	651 (1-29,241)	33 (0.072-1,426)	98 (0.427-867)	426 (1-20,734)	23 (0.060-1,111)
**Burkina Faso**	21 (0.740-112)	103 (2-3,076)	5 (0.073-147)	17 (0.701-88)	77 (1-2,474)	4 (0.088-121)
**Burundi**	289 (41-611)	1,350 (42-28,991)	63 (2-1,341)	209 (29-482)	979 (35-20,908)	48 (2-1,009)
**Cameroon**	144 (0.075-592)	556 (0-22,383)	25 (0.015-1,032)	108 (0.029-460)	390 (0-16,860)	19 (0.007-795)
**Central African Republic**	29 (0.055-248)	127 (0-6,180)	5 (0.009-257)	22 (0.070-176)	90 (0-4,065)	4 (0.012-161)
**Chad**	53 (13-150)	278 (12-5,640)	11 (0.495-238)	36 (9-111)	189 (7-3,715)	8 (0.419-162)
**Comoros**	1 (0.893-2)	8 (0-123)	0.384 (0.017-6)	1 (0.576-2)	5 (0-95)	0.285 (0.019-5)
**Congo Rep**	14 (0.046-105)	62 (0-2,648)	3 (0.005-130)	10 (0.029-84)	49 (0-2,417)	3 (0.005-124)
**Cote dIvoire**	225 (0.106-1,216)	742 (0-26,090)	32 (0.011-1,165)	148 (0.095-883)	448 (0-18,134)	21 (0.013-808)
**Dem Republic of Congo**	2,641 (451-5,439)	12,503 (456-284,532)	590 (21-13,047)	1,707 (298-4,062)	8,062 (286-170,436)	403 (19-8,376)
**Djibouti**	2 (1-3)	10 (0-169)	0.534 (0.024-9)	1 (0.626-2)	6 (0-106)	0.350 (0.020-6)
**Eritrea**	43 (17-83)	228 (10-3,998)	12 (0.474-199)	32 (12-64)	164 (7-3,114)	9 (0.514-167)
**Ethiopia**	877 (305-1,726)	11,453 (464-202,353)	630 (24-11,262)	560 (194-1,196)	7,185 (344-134,925)	417 (22-7,718)
**Gambia**	9 (0.003-39)	35 (0-1,615)	2 (0.000-77)	7 (0.002-30)	25 (0-970)	1 (0.000-48)
**Ghana**	15 (0.582-75)	67 (1-1,996)	3 (0.042-94)	10 (0.498-55)	50 (1-1,551)	3 (0.051-76)
**Guinea**	82 (0.199-516)	347 (1-12,862)	16 (0.035-582)	55 (0.125-349)	225 (0-7,629)	11 (0.026-387)
**Guinea Bissau**	4 (0.013-33)	17 (0-821)	0.694 (0.002-33)	3 (0.011-23)	12 (0-587)	0.553 (0.001-26)
**Kenya**	209 (111-351)	1,660 (99-24,032)	81 (5-1,191)	151 (72-270)	1,266 (78-17,879)	65 (4-913)
**Lesotho**	0.343 (0.008-2)	2 (0-58)	0.062 (0.001-2)	0.244 (0.006-2)	1 (0-46)	0.046 (0.001-2)
**Liberia**	83 (0.048-572)	340 (0-15,688)	16 (0.007-754)	61 (0.019-432)	232 (0-11,530)	12 (0.003-565)
**Madagascar**	434 (82-899)	2,119 (67-48,811)	111 (3-2,587)	299 (55-667)	1,353 (55-31,431)	75 (4-1,708)
**Malawi**	20 (0.706-96)	96 (1-2,633)	5 (0.053-135)	14 (0.418-74)	69 (1-1,868)	4 (0.049-98)
**Mali**	37 (12-91)	191 (8-3,586)	9 (0.342-166)	26 (8-71)	134 (6-2,790)	7 (0.371-136)
**Mauritania**	6 (2-12)	29 (1-560)	1 (0.059-29)	4 (1-10)	21 (1-389)	1 (0.067-20)
**Mozambique**	55 (2-269)	257 (4-8,293)	11 (0.182-379)	41 (2-206)	185 (3-5,852)	9 (0.189-267)
**Niger**	88 (0.259-582)	383 (1-16,045)	19 (0.033-831)	67 (0.391-439)	285 (1-11,324)	15 (0.062-565)
**Nigeria**	1,384 (0-11,316)	19,116 (0-852,033)	749 (0-32,223)	992 (0-7,632)	11,780 (0-584,308)	485 (0-24,253)
**Rwanda**	66 (30-120)	337 (15-6,979)	18 (0.773-367)	49 (20-101)	255 (11-4,718)	14 (0.833-259)
**Sao Tome e Principe**	0.218 (0.001-2)	1 (0-39)	0.051 (0.000-2)	0.170 (0.001-1)	1 (0-31)	0.039 (0.000-2)

**Senegal**	63 (0.012-272)	173 (0-4,963)	9 (0.002-256)	49 (0.028-212)	123 (0-3,905)	7 (0.005-217)
**Sierra Leone**	90 (0.033-543)	355 (0-13,405)	14 (0.006-544)	74 (0.020-420)	293 (0-11,683)	13 (0.003-482)
**Somalia**	100 (53-179)	549 (24-9,411)	22 (0.917-393)	67 (31-133)	354 (15-6,388)	15 (0.931-267)
**South Sudan**	63 (36-103)	329 (15-5,530)	14 (0.664-243)	40 (19-72)	199 (9-3,648)	9 (0.604-166)
**Tanzania**	313 (2-2,081)	1,383 (5-59,000)	74 (0.299-3,128)	229 (1-1,502)	966 (4-36,354)	55 (0.248-2,059)
**Togo**	41 (0.015-168)	159 (0-5,938)	8 (0.002-288)	33 (0.013-139)	122 (0-4,617)	6 (0.003-227)
**Uganda**	125 (82-182)	689 (31-11,752)	33 (1-568)	94 (52-147)	490 (22-9,121)	25 (2-453)
**Zambia**	32 (1-164)	149 (2-4,643)	7 (0.104-216)	25 (0.768-135)	120 (1-3,603)	6 (0.080-180)
**Zimbabwe**	14 (0.269-114)	103 (1-3,139)	5 (0.033-143)	10 (0.192-79)	72 (1-2,581)	3 (0.037-119)
**Sub-Saharan Africa Total**	**9,905 (5,104-19,497)**	**99,924 (6,240-1,359,231)**	**4,546 (344-59,198)**	**6,819 (3,222-13,835)**	**65,762 (4,146-897,615)**	**3,093 (239-41,081)**
North Africa & the Middle East						
**Afghanistan**	1,980 (289-3,747)	9,015 (248-202,260)	453 (13-10,129)	1,302 (190-2,746)	5,821 (185-120,656)	312 (12-6,349)
**Republic of Sudan**	151 (51-292)	789 (31-15,321)	42 (2-837)	108 (36-232)	525 (22-10,191)	30 (2-579)
**Yemen**	389 (33-1,086)	1,844 (60-40,017)	103 (3-2,284)	217 (16-659)	957 (21-24,369)	57 (1-1,383)
**North Africa and Middle East Total**	**2,575 (745-4,439)**	**11,501 (491-229,431)**	**625 (36-11919)**	**1,654 (425-3209)**	**7,626 (300-150,056)**	**51 (425-3,209)**
Central Europe, Eastern Europe, & Central Asia						
**Armenia**	2 (0.180-4)	9 (0-209)	0.484 (0.015-12)	1 (0.105-3)	5 (0-102)	0.305 (0.012-6)
**Azerbaijan**	18 (1-36)	77 (2-1,584)	4 (0.090-81)	11 (0.757-27)	48 (1-1,125)	3 (0.076-62)
**Georgia**	2 (0.224-4)	10 (0-212)	0.548 (0.016-11)	1 (0.118-3)	5 (0-109)	0.324 (0.011-6)
**Kyrgyz Republic**	6 (0.370-11)	23 (1-552)	1 (0.039-30)	4 (0.231-9)	15 (0-357)	0.915 (0.027-20)
**Moldova**	1 (0.069-2)	5 (0-109)	0.269 (0.005-6)	0.645 (0.039-2)	3 (0-62)	0.159 (0.004-4)
**Tajikistan**	10 (0.895-21)	43 (1-952)	2 (0.066-52)	7 (0.662-16)	30 (1-664)	2 (0.062-39)
**Ukraine**	15 (1-28)	65 (2-1,293)	3 (0.078-69)	8 (0.607-18)	35 (1-836)	2 (0.064-46)
**Uzbekistan**	27 (2-57)	117 (3-2,649)	6 (0.165-135)	17 (1-42)	74 (2-1,745)	4 (0.121-92)
**Central Europe, Eastern Europe, and Central Asia Total**	**80 (38-126)**	**386 (19-6,656)**	**21 (1-347)**	**51 (22-91)**	**248 (11-4,185)**	**14 (1-232)**
South Asia						
**Bangladesh**	12,083 (7,550-16,992)	25,746 (401-1,422,299)	1,542 (24-87,212)	7,904 (3,951-12,650)	18,305 (220-856,253)	1,191 (41-52,628)
**Bhutan**	17 (7-28)	90 (4-1,629)	5 (0.206-93)	10 (3-19)	49 (2-919)	3 (0.166-54)
**India**	36,643 (11,373-62,790)	359,802 (61,235-1,741,923)	20,187 (3,412-98,845)	21,090 (6,256-41,256)	219,842 (37,559-1,035,058)	12,959 (2,186-59,841)
**Nepal**	658 (275-1,099)	3,438 (142-60,025)	203 (8-3,615)	438 (165-808)	2,163 (105-40,404)	135 (8-2,477)
**Pakistan**	2,704 (819-5,135)	5,141 (477-52,967)	285 (27-2,928)	1,555 (436-3,195)	3,215 (264-33,271)	199 (20-1,901)
**South Asia Total**	**52,575 (25,997-79,586)**	**470,454 (95,359-2,617,226)**	**26,902 (5,511-146,150)**	**31,183 (13,581-53,439)**	**280,540 (52,721-1,595,374)**	**16,567 (3,215-94,710)**
Southeast Asia, East Asia, & Oceania						
**Cambodia**	568 (199-948)	2,952 (117-53,605)	167 (7-3,093)	374 (113-692)	1,794 (80-35,749)	110 (7-2,084)

**DPR Korea**	130 (8-458)	592 (13-15,038)	32 (0.705-852)	76 (6-315)	360 (9-9,065)	21 (0.641-533)
**Indonesia**	36 (2-236)	79 (3-1,083)	4 (0.158-59)	21 (0.792-144)	42 (1-538)	3 (0.083-33)
**Kiribati**	2 (0.147-6)	9 (0-218)	0.498 (0.012-12)	1 (0.081-4)	5 (0-141)	0.319 (0.010-8)
**Lao PDR**	191 (12-488)	259 (5-9,129)	14 (0.262-482)	120 (9-339)	166 (3-5,715)	10 (0.305-325)
**Mongolia**	47 (3-101)	206 (4-4,953)	10 (0.215-245)	29 (2-74)	128 (3-2,866)	7 (0.200-160)
**Myanmar**	709 (45-1,477)	3,245 (64-73,774)	169 (4-3,883)	409 (26-989)	1,704 (42-44,353)	94 (3-2,465)
**Papua New Guinea**	674 (41-1,481)	2,848 (70-65,753)	153 (4-3,362)	319 (15-925)	1,398 (29-33,528)	81 (2-1,904)
**Solomon Islands**	19 (1-68)	87 (2-2,591)	5 (0.103-151)	14 (0.760-54)	66 (1-1,718)	4 (0.086-104)
**Sri Lanka**	163 (37-287)	792 (31-14,716)	46 (2-944)	96 (22-202)	453 (18-9,071)	28 (1-574)
**Timor Leste**	21 (2-44)	98 (3-2,037)	6 (0.167-121)	15 (0.962-34)	66 (1-1,426)	4 (0.108-86)
**Vietnam**	478 (34-1,138)	8,906 (405-106,431)	534 (24-6,279)	268 (18-783)	5,383 (210-62,487)	341 (13-4,015)
**Southeast Asia, East Asia, and Oceania Total**	**3,141 (1,766-4,668)**	**25,919 (2,461-268,270)**	**1,495 (151-14,714)**	**1,875 (930-3,106)**	**15,624 (1,366-163,411)**	**940 (90-9,661)**
Latin America & the Caribbean						
**Bolivia**	19 (0.987-91)	94 (2-2,366)	5 (0.088-127)	13 (0.706-63)	62 (1-1,675)	4 (0.067-93)
**Cuba**	2 (0.089-8)	8 (0-198)	0.476 (0.009-12)	0.935 (0.062-5)	5 (0-122)	0.290 (0.006-8)
**Guyana**	0.571 (0.042-3)	3 (0-76)	0.132 (0.003-4)	0.329 (0.021-2)	2 (0-54)	0.085 (0.002-3)
**Haiti**	12 (0.842-53)	57 (1-1,510)	2 (0.049-68)	7 (0.474-33)	33 (1-909)	2 (0.044-42)
**Honduras**	5 (0.535-24)	27 (1-750)	2 (0.035-43)	4 (0.301-17)	18 (0-532)	1 (0.026-32)
**Nicaragua**	5 (0.310-22)	22 (1-632)	1 (0.030-38)	3 (0.201-16)	15 (0-378)	0.908 (0.024-23)
**Latin America and Caribbean Total**	**54 (19-127)**	**263 (13-5130)**	**14 (1-267)**	**34 (11-88)**	**166 (8-3313)**	**9 (1-186)**
**TOTAL**	**69,658 (41,963-96,898)**	**848,553 (308,302-3,072,196)**	**45,090 (17,795-172,577)**	**42,515 (24,795-62,895)**	**506,026 (186,661-1,962,678)**	**27,923 (9,694-126,302)**
**Percent of burden averted**	--	--	--	**66.5% (49.2%-79.2%)**	**67.3% (50.1%-76.9%)**	**70.5% (50.0%-97.1%)**

Table S8. Baseline burden and vaccine impact on MDR typhoid fever. Results are for a 10-year time horizon. Countries are grouped by GBD super-region.

Country	Baseline MDR cases (thousands) (95% prediction interval)	Baseline MDR deaths (95% prediction interval)	Baseline MDR DALYs (thousands) (95% prediction interval)	MDR cases averted (thousands) (95% prediction interval)	MDR deaths averted (95% prediction interval)	MDR DALYs averted (thousands) (95% prediction interval)
Sub-Saharan Africa						
**Angola**	90 (0-800)	256 (0-23,398)	10 (0-914)	62 (0-587)	181 (0-15,838)	8 (0-634)
**Benin**	345 (18-1,528)	1,764 (31-43,809)	88 (2-2,263)	243 (13-1,120)	1,213 (23-30,780)	65 (1-1,575)
**Burkina Faso**	22 (0.677-117)	105 (2-3,219)	5 (0.075-153)	16 (0.443-89)	78 (1-2,332)	4 (0.067-111)
**Burundi**	702 (100-1,538)	3,289 (117-60,833)	153 (5-2,917)	506 (72-1,214)	2,391 (85-46,117)	117 (6-2,226)
**Cameroon**	94 (0-642)	276 (0-25,023)	13 (0 -1,167)	63 (0-517)	200 (0-19,401)	11 (0-906)
**Central African Republic**	75 (5-304)	363 (9-10,018)	14 (0.332-394)	50 (3-219)	250 (5-6,020)	11 (0.279-251)
**Chad**	71 (11-240)	361 (12-8,760)	15 (0.495-354)	49 (7-176)	248 (8-6,166)	11 (0.441-259)
**Comoros**	3 (2-5)	19 (1-275)	0.944 (0.047-14)	3 (1-4)	14 (1-221)	0.759 (0.047-11)
**Congo Rep**	31 (2-130)	160 (3-3,643)	8 (0.139-184)	23 (1-101)	119 (2-2,979)	6 (0.134-148)
**Cote d'Ivoire**	186 (0-1,335)	476 (0-31,432)	20 (0-1,329)	130 (0-1,030)	328 (0-21,004)	16 (0-947)
**Dem Republic of Congo**	2,559 (439-5,237)	12,200 (468-245,557)	569 (22-11,050)	1,695 (283-3,816)	8,181 (298-165,715)	408 (18-7,812)
**Djibouti**	5 (3-7)	26 (1-355)	1 (0.066-19)	3 (1-5)	17 (1-247)	0.889 (0.062-13)
**Eritrea**	105 (40-203)	549 (28-8,436)	28 (1-457)	77 (28-162)	408 (21-6,465)	22 (1-354)
**Ethiopia**	2,152 (732-4,248)	28,479 (1,604-510,994)	1,562 (87-27,365)	1,356 (438-2,932)	18,457 (935-339,273)	1,059 (56-18,414)
**Gambia**	6 (0-43)	17 (0-1,554)	0.821 (0-76)	5 (0.000-35)	13 (0-1,181)	0.692 (0-59)
**Ghana**	392 (272-510)	2,216 (114-33,621)	107 (6-1,584)	297 (170-430)	1,665 (82-26,805)	86 (5-1,313)
**Guinea**	177 (12-609)	816 (23-18,748)	39 (1-898)	123 (8-447)	579 (15-12,651)	29 (0.949-624)
**Guinea Bissau**	9 (0.466-36)	42 (1-1,215)	2 (0.042-52)	6 (0.341-28)	31 (1-924)	1 (0.040-39)
**Kenya**	762 (610-936)	6,379 (403-79,116)	315 (19-3,745)	570 (357-776)	4,674 (298-60,578)	243 (17-3,053)
**Lesotho**	2 (0.191-7)	10 (0-210)	0.373 (0.011-8)	1 (0.130-5)	7 (0-146)	0.275 (0.011-6)
**Liberia**	49 (0-640)	164 (0-20,126)	8 (0-934)	36 (0-494)	119 (0-14,265)	6 (0-696)
**Madagascar**	1,040 (204-2,245)	4,992 (207-89,751)	262 (11-4,870)	727 (141-1,681)	3,396 (139-64,768)	188 (9-3,481)
**Malawi**	97 (19-224)	485 (19-8,273)	25 (0.980-420)	71 (14-177)	353 (14-6,064)	19 (1-322)
**Mali**	48 (8-148)	252 (9-4,779)	12 (0.431-225)	35 (6-114)	181 (7-3,702)	9 (0.430-177)
**Mauritania**	7 (1-22)	40 (1-722)	2 (0.060-36)	5 (0.866-16)	29 (1-550)	2 (0.057-28)
**Mozambique**	280 (54-636)	1,427 (48-26,222)	62 (2-1,153)	207 (37-505)	1,073 (36-19,984)	49 (2-906)
**Niger**	220 (12-679)	1,045 (21-22,540)	53 (1-1,102)	161 (8-506)	742 (14-16,767)	40 (0.871-885)
**Nigeria**	8,325 (4,222-13,053)	157,966 (11,355-1,735,476)	6,251 (461-69,265)	5,183 (2,351-9,279)	98,105 (7,371-1,116,762)	3,985 (341-45,220)
**Rwanda**	162 (68-302)	853 (41-13,683)	44 (2-707)	120 (48-242)	657 (32-10,799)	35 (2-565)
**Sao Tome e Principe**	0.494 (0.026-2)	2 (0-54)	0.126 (0.002-3)	0.365 (0.019-1)	2 (0-40)	0.103 (0.002-2)
**Senegal**	42 (0.000-307)	101 (0-6,674)	5 (0.000-354)	31 (0.000-244)	76 (0-4,895)	4 (0.000-283)

**Sierra Leone**	67 (0.000-598)	204 (0-19,131)	8 (0.000-764)	50 (0.000-468)	159 (0-14,953)	7 (0.000-599)
**Somalia**	246 (123-460)	1,361 (66-21,653)	55 (3-875)	165 (72-336)	927 (42-15,115)	40 (3-644)
**South Sudan**	150 (80-252)	851 (40-11,838)	36 (2-523)	97 (44-179)	526 (25-8,390)	24 (2-379)
**Tanzania**	804 (302-1,559)	4,263 (189-72,209)	228 (10-3,755)	597 (208-1,247)	3,204 (138-55,953)	178 (10-2,993)
**Togo**	27 (0.000-185)	83 (0-7,720)	4 (0.000-365)	21 (0.000-154)	62 (0-5,439)	3 (0.000-269)
**Uganda**	303 (174-448)	1,693 (85-26,336)	81 (4-1,263)	230 (116-370)	1,239 (63-19,441)	63 (4-988)
**Zambia**	161 (28-427)	831 (32-14,207)	40 (1-677)	120 (22-338)	632 (22-11,231)	32 (1-568)
**Zimbabwe**	81 (5-287)	612 (14-11,733)	28 (0.651-518)	59 (3-228)	464 (12-8,703)	21 (0.581-413)
**Sub-Saharan Africa Total**	**21,179 (15,122-28,696)**	**263,515 (24,314-2,947,403)**	**11,951 (1,195-131,340)**	**14,392 (8,406-21,524)**	**173,735 (15,808-2,024,517)**	**8,019 (802-94,770)**
North Africa & the Middle East						
**Afghanistan**	79 (11-227)	388 (13-8,376)	20 (0.675-426)	52 (7-159)	254 (9-5,559)	14 (0.530-294)
**Republic of Sudan**	371 (127-734)	1,907 (88-34,283)	102 (5-1,852)	263 (88-574)	1,418 (63-26,549)	81 (4-1,420)
**Yemen**	482 (36-1,817)	2,292 (57-60,544)	127 (3-3,367)	257 (17-1,095)	1,176 (26-34,837)	71 (2-2,087)
**North Africa & Middle East Total**	**987 (370-2,233)**	**5,000 (227-105,077)**	**278 (16-5,950)**	**625 (207-1,506)**	**3,141 (136-67,758)**	**183 (10-3,880)**
Central Europe, Eastern Europe, & Central Asia						
**Armenia**	0.780 (0.059-3)	4 (0-95)	0.215 (0.004-5)	0.436 (0.031-2)	2 (0-55)	0.136 (0.003-3)
**Azerbaijan**	7 (0.290-25)	31 (1-867)	2 (0.034-46)	4 (0.176-17)	20 (0-580)	1 (0.027-31)
**Georgia**	0.853 (0.059-3)	4 (0-104)	0.227 (0.004-5)	0.455 (0.031-2)	2 (0-54)	0.136 (0.003-3)
**Kyrgyz Republic**	2 (0.102-8)	10 (0-226)	0.563 (0.010-12)	1 (0.063-6)	7 (0-152)	0.405 (0.008-9)
**Moldova**	0.421 (0.016-2)	2 (0-49)	0.107 (0.001-3)	0.249 (0.009-1)	1 (0-30)	0.067 (0.001-2)
**Tajikistan**	10 (0.982-23)	47 (2-934)	3 (0.081-51)	7 (0.739-18)	35 (1-682)	2 (0.082-38)
**Ukraine**	5 (0.327-21)	26 (0-606)	1 (0.024-30)	3 (0.173-13)	15 (0-356)	0.837 (0.020-20)
**Uzbekistan**	4 (0.331-10)	19 (0-381)	0.924 (0.024-20)	3 (0.191-7)	12 (0-250)	0.638 (0.020-13)
**Central Europe, Eastern Europe, & Central Asia Total**	**35 (15-62)**	**165 (9-3298)**	**9 (1-170)**	**22 (9-44)**	**108 (6-2224)**	**6 (0-121)**
South Asia						
**Bangladesh**	4,176 (2,711-5,942)	9,712 (162-427,120)	591 (10-26,734)	2,781 (1,385-4,430)	6,565 (100-278,667)	435 (18-17,807)
**Bhutan**	0.707 (0.224-2)	4 (0-69)	0.214 (0.008-4)	0.410 (0.115-1)	2 (0-41)	0.132 (0.007-2)
**India**	925 (233-2,601)	9,353 (1,337-60,880)	531 (74-3,375)	538 (131-1,626)	5,561 (794-36,255)	324 (47-2,109)
**Nepal**	10 (2-29)	53 (2-1,034)	3 (0.126-62)	7 (2-20)	35 (1-713)	2 (0.112-43)
**Pakistan**	2,071 (1,164-3,215)	4,262 (497-39,149)	237 (28-2,302)	1,177 (575-2,077)	2,473 (271-23,210)	150 (20-1,371)
**South Asia Total**	**7,288 (5,142-9,881)**	**32,532 (5,436-465,482)**	**1,903 (340-28,551)**	**4,609 (2,615-6,907)**	**19,642 (2,992-309,720)**	**1,197 (203-19,081)**
Southeast Asia, East Asia, & Oceania						
**Cambodia**	485 (223-833)	2,719 (123-40,131)	154 (7-2,223)	314 (136-619)	1,714 (79-28,709)	102 (6-1,681)

**DPR Korea**	14 (0.323-111)	71 (1-2,382)	4 (0.032-134)	8 (0.199-71)	42 (0-1,531)	2 (0.025-89)
**Indonesia**	72 (3-474)	154 (5-1,821)	9 (0.252-101)	42 (2-276)	89 (2-1,058)	6 (0.159-63)
**Kiribati**	1 (0.013-5)	5 (0-168)	0.296 (0.002-9)	0.723 (0.008-4)	4 (0-115)	0.206 (0.001-7)
**Lao PDR**	130 (1-470)	168 (1-6,870)	9 (0.047-379)	83 (0.900-332)	106 (1-4,557)	7 (0.044-263)
**Mongolia**	18 (0.687-68)	89 (2-2,129)	4 (0.083-108)	11 (0.419-46)	54 (1-1,392)	3 (0.065-75)
**Myanmar**	448 (6-1,411)	1,816 (13-57,231)	94 (0.673-2,918)	266 (3-918)	1,074 (8-33,041)	61 (0.522-1,882)
**Papua New Guinea**	411 (4-1,456)	1,907 (10-50,131)	102 (0.527-2,762)	204 (2-878)	951 (4-27,077)	56 (0.229-1,488)
**Solomon Islands**	12 (0.152-63)	54 (0-1,777)	3 (0.026-109)	9 (0.112-48)	39 (0-1,415)	2 (0.022-85)
**Sri Lanka**	6 (1-18)	32 (1-614)	2 (0.069-36)	4 (0.760-12)	19 (1-387)	1 (0.056-24)
**Timor Leste**	13 (0.144-42)	60 (1-1,510)	4 (0.031-93)	10 (0.108-32)	43 (0-1,125)	3 (0.023-68)
**Vietnam**	311 (2-1,143)	5,975 (56-78,733)	360 (3-4,636)	184 (2-758)	3,561 (32-49,185)	222 (2-3,113)
**Southeast Asia, East Asia, and Oceania Total**	**2,220 (990-3,787)**	**18,097 (1,430-214,280)**	**1,038 (89-12,367)**	**1,331 (541-2,424)**	**10,737 (814-138,561)**	**638 (54-8,334)**
Latin America & the Caribbean						
**Bolivia**	6 (0.189-43)	28 (0-923)	1 (0.019-48)	4 (0.121-30)	18 (0-646)	1 (0.015-35)
**Cuba**	0.526 (0.014-3)	2 (0-93)	0.138 (0.002-5)	0.283 (0.008-2)	1 (0-51)	0.082 (0.001-3)
**Guyana**	0.175 (0.004-1)	1 (0-28)	0.041 (0.001-1)	0.099 (0.002-0.722)	0 (0-17)	0.027 (0.000-0.907)
**Haiti**	4 (0.119-25)	18 (0-657)	0.767 (0.012-28)	2 (0.062-17)	10 (0-385)	0.492 (0.009-18)
**Honduras**	2 (0.063-12)	8 (0-278)	0.466 (0.007-16)	1 (0.039-8)	5 (0-186)	0.335 (0.006-11)
**Nicaragua**	1 (0.049-10)	7 (0-227)	0.394 (0.005-13)	0.919 (0.031-6)	4 (0-155)	0.282 (0.004-9)
**Latin America & Caribbean Total**	**18 (5-57)**	**94 (4-1898)**	**5 (0-97)**	**12 (3-39)**	**58 (2-1196)**	**3 (0-63)**
**TOTAL**	**32,236 (25,506-38,951)**	**479,108 (202,192-2,163,659)**	**24,866 (9,716-97,530)**	**21,218 (16,410-26,467)**	**342,725 (135,506-1,573,981)**	**16,508 (6,975-88,386)**
**Percent of burden averted**	--	--	--	**67.4% (50.0-90.4%)**	**66.3% (49.2%-79.3%)**	**69.2% (50%-87.6%)**

7. Supplementary references

1. Bilcke J, Antillón M, Pieters Z, et al. Cost-effectiveness of routine and campaign use of typhoid Vi-conjugate vaccine in Gavi-eligible countries: a modelling study. Lancet Infect Dis 2019; 19: 728–39.

2. United Nations. UN World Population Prospects. https://esa.un.org/unpd/wpp/DataQuery/.

3. Hornick RB, Greisman SE, Woodward TE, Dupont HL, Dawkins AT, Snyder MJ. Typhoid Fever: Pathogenesis and Immunologic Control. N. Engl. J. Med. 1970; 283: 686–91.

4. Ames WR, Robins M. Age and Sex as Factors in the Development of the Typhoid Carrier State, and a Method for Estimating Carrier Prevalence. Am J Public Heal Nations Heal 1943; 33: 221–30.

5. Antillón M, Bilcke J, Paltiel AD, Pitzer VE. Cost-effectiveness analysis of typhoid conjugate vaccines in five endemic low- and middle-income settings. Vaccine 2017; 35: 3506–14.

6. Jin C, Gibani MM, Moore M, et al. Efficacy and immunogenicity of a Vi-tetanus toxoid conjugate vaccine in the prevention of typhoid fever using a controlled human infection model of Salmonella Typhi: a randomised controlled, phase 2b trial. Lancet 2017; 390: 2472–80.

7. Voysey M, Pollard AJ. Seroefficacy of Vi Polysaccharide–Tetanus Toxoid Typhoid Conjugate Vaccine (Typbar TCV). Clin Infect Dis 2018; 67: 18–24.

8. Pitzer VE, Pollard AJ, Bilcke J. Strategies for typhoid conjugate vaccines in endemic nations – Authors' reply. Lancet Infect. Dis. 2021; 21: 321–2.

9. Shakya M, Colin-Jones R, Theiss-Nyland K, et al. Phase 3 efficacy analysis of a typhoid conjugate vaccine trial in Nepal. N Engl J Med 2019; 381: 2209–18.

10. Lanh MN, Van Bay P, Ho VA, et al. Persistent Efficacy of Vi Conjugate Vaccine against Typhoid Fever in Young Children. N Engl J Med 2003; 349: 1390–1.

11. Stanaway JD, Reiner RC, Blacker BF, et al. The global burden of typhoid and paratyphoid fevers: a systematic analysis for the Global Burden of Disease Study 2017. Lancet Infect Dis 2019; 19: 369–81.

12. Antillón M, Warren JL, Crawford FW, et al. The burden of typhoid fever in low- and middle-income countries: A meta-regression approach. PLoS Negl Trop Dis 2017; 11. DOI:10.1371/journal.pntd.0005376.

13. Browne AJ, Kashef Hamadani BH, Kumaran EAP, et al. Drug-resistant enteric fever worldwide, 1990 to 2018: A systematic review and meta-analysis. BMC Med 2020; 18: 1–22.

14. Britto CD, Wong VK, Dougan G, Pollard AJ. A systematic review of antimicrobial resistance in Salmonella enterica serovar Typhi, the etiological agent of typhoid. PLoS Negl Trop Dis 2018; 12: 1–15.

15. Joseph L, Bélisle P. beta.parms.from.quantiles: [R] Computing Beta distribution parameters. 2017. http://www.medicine.mcgill.ca/epidemiology/Joseph/PBelisle/BetaParmsFromQuantiles.html (accessed March 13, 2021).

16. Rahman BA, Wasfy MO, Maksoud MA, Hanna N, Dueger E, House B. Multi-drug resistance and reduced susceptibility to ciprofloxacin among Salmonella enterica serovar Typhi isolates from the Middle East and Central Asia. New Microbes New Infect 2014; 2: 88–92.

17. Lynch MF, Blanton EM, Bulens S, et al. Typhoid fever in the United States, 1999-2006. JAMA - J Am Med Assoc 2009; 302: 859–65.

18. Bano-Zaidi M, Aguayo-Romero M, Campos FD, et al. Typhoid fever outbreak with severe complications in Yucatan, Mexico. Lancet Glob Heal 2018; 6: e1062–3.

19. Diaz-Guevara P, Montaño LA, Duarte C, et al. Surveillance of salmonella enterica serovar typhi in Colombia, 2012–2015. PLoS Negl Trop Dis 2020; 14: 2012–5.

20. Wu W, Wang H, Lu J, et al. Genetic Diversity of Salmonella enteric serovar Typhi and Paratyphi in Shenzhen, China from 2002 through 2007. BMC Microbiol 2010; 10. DOI:10.1186/1471-2180-10-32.

21. Pitzer VE, Bowles CC, Baker S, et al. Predicting the impact of vaccination on the transmission dynamics of typhoid in South Asia: a mathematical modeling study. PLoS Negl Trop Dis 2014; 8: e2642.

22. Crump JA, Luby SP, Mintz ED. The global burden of typhoid fever. Bull World Health Organ 2004; 82: 346–53.

23. Pieters Z, Saad NJ, Antillón M, Pitzer VE, Bilcke J. Case Fatality Rate of Enteric Fever in Endemic Countries: A Systematic Review and Meta-analysis. Clin Infect Dis An Off Publ Infect Dis Soc Am 2018; 67: 628.
